# Genomic insights into the functional and metabolic versatility of gut microbiome Anaerostipes species

**DOI:** 10.1099/mgen.0.001617

**Published:** 2026-02-09

**Authors:** Disha Bhattacharjee, Lindsey C. Millman, Meagan L. Seesengood, Lindsey M. Martineau, Anna M. Seekatz

**Affiliations:** 1 Department of Biological Sciences, Clemson University, Clemson, South Carolina, USA

**Keywords:** *Anaerostipes*, bacterial cultivation, *Clostridia*, functional pathways, genomics, gut commensal

## Abstract

Members of the class *Clostridia*, a polyphyletic group of pathogenic and beneficial Gram-positive, spore-forming anaerobes in the *Bacillota* (Firmicutes) phylum, are prevalent in the human gut. While this class includes select pathogens known to cause disease, many species are associated with beneficial functions, such as providing colonization resistance against pathogens. Despite a demonstrated value in maintaining Clostridial populations in the gut, functional strain diversity of most commensal Clostridial species remains understudied. Here, we isolated and characterized Clostridial isolates, focusing on the genomic diversity of *Anaerostipes*, a prevalent butyrate-producing genus within the gut microbiota. We conducted a genomic comparison across 21 *Anaerostipes* strains isolated from healthy human faecal samples (*n*=5) and publicly available genomes (*n*=105). Whole genome comparisons across the *Anaerostipes* genus demonstrated 12 species bins, clustering into three major functionally distinct clusters correlating with host origin. One cluster (representing mostly *Anaerostipes caccae* genomes) was distinguished by possessing a complete vitamin B12 biosynthesis pathway. Variability in genomic and phenotypic carbohydrate metabolism was demonstrated within dominant species of the human microbiota (*Anaerostipes hadrus*, *A. caccae* and *Anaerostipes hominis*). Collectively, these data indicate genomic metabolic variance across *Anaerostipes* species that may influence coexistence within the gut environment and variably influence health.

## Data Summary

All raw sequence data and associated information have been deposited in the NCBI Sequence Read Archive under BioProject PRJNA1241798. All code used to analyse data is available at https://github.com/SeekatzLab/Anaerostipes-genomes.

**Impact Statement**

*Anaerostipes* is a bacterial genus containing species known to produce butyrate upon fermenting lactate. Despite their association with health across studies using 16S rRNA gene-based analyses, little is known about genomic variability within and across species. Our study aims to define strain variability across the *Anaerostipes* genus, identifying three functional clusters and close phylogenetic distance within species found in the human gut. Major variability within species prevalent in the human gut included variable carbohydrate and amino acid metabolism genes, suggesting the ability to coexist in the gut environment.

## Introduction

The gut microbiota plays a vital role in human health by providing essential physiological and developmental functions, including training host immunity, supporting digestion of dietary intake and regulating neurological signalling [1]. Disruption of this ecosystem can lead to colonization of pathogenic bacteria or loss of species that support these functions, influencing various conditions or diseases that harm the host [2]. Understanding the tenets that uphold the structure of a healthy or beneficial microbiota, both at the community and individual species level, can thus guide preventative and interventional methods to modulate the gut microbiota, which may ultimately be used to treat disease.

In addition to considering how differences in species composition influence gut microbiota functions, genotypic variation across individual strains may influence microbial functional outcomes [3]. Much of our initial definition of a healthy microbiota has been based on 16S rRNA gene-based analyses, which cannot typically distinguish taxonomic groups beyond the genus level [4]. While useful for initial surveys of microbiota, this method cannot account for gene content or genomic differences within a given species and can disparately influence the overall output of a community or differentially impact the host [5,7]. Although genomic variation is well-established when considering virulence of pathogens [8], its importance has been less considered within the context of commensal gut species.

Lack of information on phenotypic and genomic variation is especially true for many members of the polyphyletic class *Clostridia* [9], which encompasses spore-forming, saprophytic anaerobes. Many species within this group, such as *Faecalibacterium prausnitzii*, *Clostridium scindens*, *Coprococcus comes*, *Clostridium sporogenes* and others, are also frequently correlated with health [10]. Importantly, this group includes many species that produce metabolites of interest to human health. *Lachnospiraceae*, such as *Coprococcus*, *Roseburia* and *Anaerostipes*, are known to produce short-chain fatty acids (SCFAs) upon fermentation of dietary fibre and resistant starches [11,14]. The SCFA, butyrate, is a main energy source for colonocytes and has been demonstrated to play a regulatory role in epithelial defence barriers, intestinal motility and inflammation, making microbes producing butyrate promising candidates for future probiotics [15,16]. *Clostridia* also include species such as *C. scindens* and *Clostridium hiranonis* that modulate bile acids (BAs) initially produced by the host, which influence host physiology in diarrhoea-predominant irritable bowel syndrome (IBS) and colonization resistance against *Clostridioides difficile* infections [17,18]. *Clostridia* can additionally influence host health through protein fermentation. For example, *F. prausnitzii*, a butyrate producer, can influence gut physiology in gnotobiotic mice and restore serotonin in IBS murine models [19]. Similarly, another gut symbiont, *C. sporogenes*, can produce indole propionic acid from dietary tryptophan, which can fortify the intestinal barrier [20].

Well-established butyrate producers include species within the genus *Anaerostipes* [21], a member of the *Lachnospiraceae* family belonging to the phylum *Bacillota* (previously Firmicutes) [22]. Initially classified solely as *Anaerostipes caccae*, the *Anaerostipes* genus now contains the major species *A. caccae*, *Anaerostipes hadrus*, *Anaerostipes rhamnosivorans* and *Anaerostipes butyraticus* [23,26]. Lower abundances of *Anaerostipes* have been associated with gut diseases, such as colorectal cancer [27], inflammatory bowel disease and IBS [28,29]. *A. hadrus* and other *Clostridia* have been studied *in vitro* for their ability to produce butyrate via butyryl-CoA:acetate CoA-transferase [30] and butyrate kinase [31] pathways. Most recently, *A. caccae* administration in mice was shown to be effective in reducing severe responses to allergen challenge, demonstrating a role in immune modulation and potential therapeutic response [32]. However, both genomic variability and characterization of this species’ niche within the gut microbiota in directing potential beneficial functions remain less characterized.

Here, we investigate the genomic diversity and metabolic potential of *Anaerostipes* using comparative genomics. An isolation pipeline targeting *Lachnospiraceae* species from the human gut consistently demonstrated the presence of *A. hadrus* and * A. caccae*. Genomic comparison to high-quality genomes from publicly available databases demonstrated both host-specific inter-species specialization (clustering into three distinct functional clusters) and intra-species variation across human-associated *Anaerostipes*. In addition to exhibiting widespread butyrate-producing pathways, *Anaerostipes* also exhibited genes associated with BA modification, vitamin B12 production and tocopherol cycling, expanding the potential functional contributions of *Anaerostipes* in the human gut.

## Methods

### Isolation of commensal *Clostridia* from healthy human faecal samples

This study was approved by Clemson University’s Institutional Review Board. Healthy donors were over 18, had not taken antibiotics or been diagnosed with any infections within 6 months and were not immunocompromised or diagnosed with chronic gastrointestinal conditions. Upon receipt, faecal samples were placed under anaerobic conditions (Coy Laboratory Products, Grass Lake, MI, USA; 85% nitrogen, 10% hydrogen and 5% carbon dioxide), and both a direct faecal streak and a 1:10 dilution faecal slurry were streaked out onto media typically used for anaerobic Clostridial growth: (1) brain heart infusion (BHI) supplemented with 0.5% yeast extract, (2) BHI with the addition of 5% FBS (BHI+FBS) (samples CM01–CM03) or 5% clarified, filtered rumen fluid (BHI+R) (samples CM06–CM11), (3) taurocholate cycloserine-cefoxitin-fructose agar, reinforced *Clostridia* medium and (4) yeast casitone fatty acids (YCFA) agar plates as detailed in File S1, available in the online Supplementary Material, modified from [33, 34]. The faecal slurry was also used to inoculate 5 ml of each of respective broth medium. Both the plates and broth cultures were incubated at 37 °C for 24 h. After incubation, broth cultures from media were diluted up to 10^−6^ colonies per ml and streaked out onto their respective plate types. Unique colonies based on morphology were picked from all incubated plates over the course of several days, given a unique number and re-streaked to purity. Once isolates were found to be pure, a single colony was used to inoculate 5 ml of the same media as the plate and grown for 24–48 h until visible turbidity was observed. Monoculture broths were divided into aliquots for initial taxonomic identification via Sanger sequencing and storage in 20% glycerol at −80 °C, preserving remaining broth for additional DNA extractions if necessary.

### DNA extraction and identification of faecal isolates

At initial isolation and throughout experiments, strain identity was validated using Sanger sequencing of the 16S rRNA gene. Broth aliquots were heat-extracted at 95 °C for 20 min and prepared for PCR using GoTaq (Promega; catalogue no. M7132) with the 8F (5′-AGA GTT TGA TCC TGG CTC AG-3′) and 1492R (5′-GGT TAC CTT GTT ACG ACT T-3′) primers to amplify the whole 16S rRNA gene. PCR products were cleaned up using Exo SAP-IT (Applied Biosystems, 78-200-200UL) and sent to Eton Biosciences (https://www.etonbio.com/) for Sanger sequencing. Sequences were taxonomically identified using the NCBI, EzBioCloud and RDP databases. Visualizations were done in R.

For 21 *A. hadrus* and *A. caccae* strains, DNA was extracted from 1.8 ml of overnight culture using the Qiagen DNeasy UltraClean microbial kit (Qiagen; catalogue no. 12224-250) for whole genome sequencing. Extracted DNA was diluted to 10 ng µl^−1^ concentration (Qubit, Life Technologies; catalogue no. Q33230) and sent to SeqCenter, Pittsburgh (https://www.seqcenter.com/), for Illumina sequencing using the NextSeq2000 platform.

### Assessment of *Lachnospiraceae* in human 16S rRNA gene-based surveys

Multiple FASTA sequences of full-length 16S rRNA sequences from different *Lachnospiraceae* genomes that were sequenced as a part of the isolation pipeline were formatted for alignment in mothur [35] (v1.48.0) and aligned using the SILVA database [36] (v138.2). Previously published pairs of sequences from faecal microbiota samples representing healthy adult individuals (Table S2) were processed in mothur using the Schloss lab standard operating procedure, aligning to the SILVA database and then classifying to the custom classifier using the classify.seqs command in mothur (cutoff=80) as previously published [34]. The log_10_ relative abundance percentage and prevalence of various *Lachnospiraceae* were plotted in R for visualization.

### Whole genome assembly and phylogeny

We used a previously published workflow for assembly of all genomes listed in Table S1 [34], also available on (https://github.com/SeekatzLab/Anaerostipes-genomes). Briefly, raw reads were quality-checked and adapter-trimmed using Trim-galore (v0.6.5) [37], assembled using SPAdes [38] (v3.15.5). Quast (v5.0.2) with MultiQC (v1.27.1) was used to calculate assembly statistics (Table S1) [39,40]. Average coverage was calculated using Bowtie2 and SAMtools [41,42]. Prokka (v1.14.5) was used to annotate assemblies [43]. To verify taxonomic identity, the 16S rRNA gene from assemblies were run through NCBI blast and EzBioCloud. Assemblies were also mapped on to the Genome Taxonomy Database (GTDB) [44] through GTDB-tk (v2.4.0) using classify-wf [45]. Maximum likelihood trees (unrooted) from the *Anaerostipes* core genome single nucleotide polymorphism (SNP) sites using SNP-sites (v2.5.1) were determined by Roary. Unrooted trees were created using RaXML (v8.2.12) bootstrapping 500 times [46,48]. Average amino acid identity (AAI) calculations and the resultant unweighted pair group method with arithmetic mean (UPGMA) hierarchical clustering tree were obtained using EzAAI [49]. Unrooted trees were visualized using ggtree, ggtreeExtra and treeio packages in R [50,52]. The final 126 *Anaerostipes* genomes included in analyses were limited to high-quality assemblies, with the following criteria: N50>25,000, total length between 2.5 and 3.7 Mbp, number of contigs <251, CheckM [53] contamination<5% and completeness>95%.

### Pangenome analysis, functional enrichment, average nucleotide identity and dereplication

Contigs from SPAdes were reformatted and annotated with the COG (Clusters of Orthologous Groups) and KEGG (Kyoto Encyclopedia of Genes and Genomes) databases using Anvi’o [54] (v8.0). Anvi’o was also used to create and visualize the pangenomes, determine average nucleotide identity (ANI) and dereplicate strains within the dataset. Heap’s law was calculated in R (formulated as *n*=*κN*^*γ*^, where *n* is the pangenome size, *N* is the number of genomes used and *κ* and *γ* are the fitting parameters), and the *α* parameter from Power law model was calculated using micropan [55,56]. ANI was computed using the anvi-compute-genome-similarity with pyANI [57,58]. Dereplication between strains was computed using anvi-dereplicate-genomes at 90, 95, 98, 99, 99.9 and 100% similarity threshold. Functional enrichment was calculated using anvi-compute-functional-enrichment-across-genomes along with corresponding statistics [59], and microbial metabolism is calculated using anvi-estimate-metabolism [60]. Genes for cobalamin production, sporulation and germination were identified through KEGG and COG annotation from the pangenome using Anvi’o. All visualization was performed using dplyr, ggplot2 and readxl packages [61,63].

### SCFA, BA, toxin, sporulation and germination genes and respective trees

Databases for EutD, TdcD, AbfD, Ato, Buk, But, Cro, Gcd, Kal, Epi/Mce, LcdA, MmdA, Mut, PduC, PduP, YgfH, BSH, GerAB, Spo0A, LDH, DLD and Zona protein sequences were created through UniProt [64] using the product name, bacterial taxonomy and appropriate length of the protein. DIAMOND v2.0.14 was used to create a blast appropriate database and blast (--id 70 --query-cover 80 --top 1) against proteins predicted by Prokka from each genome [65]. Visualization of the resultant hits was done in R. For GerAB, BSH, But and AckA alignments and trees, corresponding hits and their associated protein sequences were aligned using the multiple sequence aligner clustalO v1.2.4 [66]. Maximum likelihood trees (unrooted) of the resultant alignment were generated using RAxML and visualized using ggtree in R as stated above.

### CAZyme and putative virulence

Carbohydrate-active enzymes (CAZymes) were predicted using dbCAN (v4.1.4) [67], using the Fasta nucleotide sequences generated from Prokka for each of the strains and visualized through R. Prokka-generated nucleotide fasta files (.fna) were processed through PathoFact (v1.0) [68] to predict virulence factors, toxins and antimicrobial peptides and visualized through R.

### Sporulation assay

Two strains of *A. hadrus* (CM02_05 and CM03_84) and two strains of *A. caccae* (CM03_34 and CM06_64) were streaked from frozen glycerol stocks onto BHI agar plates and incubated for 48 h at 37 °C under anaerobic conditions. Plates were visually inspected for pure colony morphology, and single isolated colonies were picked and inoculated into (1) BHI for an additional 48 h of anaerobic growth for species validation using Sanger sequencing, as described above, and (2) 5 ml of pre-reduced Clospore media broth [1% w/v Special Peptone Mix (Oxoid LP0072), 1% w/v yeast extract (Sigma Y1625), 0.06% w/v ammonium sulphate (VWR EM-AX1385-1), 0.012% w/v magnesium sulphate heptahydrate (Sigma M-2773), 0.348% w/v potassium carbonate (Sigma P1472), 0.26% w/v potassium phosphate monobasic (Fisher P285)] for induction of potential sporulation, as observed with other sporulating bacteria such as *Cl. difficile* media [69,70]. These were then incubated at 37 °C for 14 days to induce sporulation. Single-phase contrast microscopy (1,000X oil immersion on a Leica DM750 Phase Contrast) was used to confirm the presence or absence of endospores, and pictures were taken with a Leica ICC50W camera and the Leica Acquire software. Sporulation assays and Sanger sequencing to validate species identity were conducted three separate times for each strain. Clospore inocula sampled at days 4 and 7 did not produce observable spores.

### *In vitro* growth assays

Four strains of *A. hadrus* (CM02_02, CM02_05, CM03_47 and CM06_29) and two strains of *A. caccae* (CM02_91A and CM06_28) were streaked from frozen glycerol stocks onto BHI agar plates and incubated at 37 °C for 48 h under anaerobic conditions (Coy Laboratory Products, Grass Lake, MI, USA; 85% nitrogen, 10% hydrogen and 5% carbon dioxide). Single isolated colonies were picked and used to inoculate 4 ml of pre-reduced BHI broth and incubated for 18 h under anaerobic conditions. To assess sugar utilization, Biolog PreBioM plates (pre-loaded carbon sources) (Biolog, F-001 and F-002) were utilized, with each containing three replicate wells per carbon source and one strain per plate. Carbon sources were resuspended with 150 µl of minimal media, basal media with amino acids (BMAA) at pH 7, as described in File S1. A Breathe-Easy cover (USA Scientific 91236100) was placed on the plate and left at room temperature for 24 h to ensure media and sugar had reduced. After incubation, the overnight culture was spun down, the supernatant was discarded and the cells were resuspended in pre-reduced ultra-pure water (Invitrogen 10977-015). After resuspension, 10 µl of cell suspension was inoculated into each well and sealed with a Breathe-Easy cover. Absorbance readings were acquired from a Tecan Sunrise Microplate reader for 24 h at 37 °C with intermittent shaking.

A similar procedure was followed for assessing strain growth in the presence of d-lactate and l-lactate, respectively. A Corning 96-well plate (Corning Inc 3595) was prepared with the following media conditions: YCFA (as described under original isolation efforts and described in File S1), basal YCFA (bYCFA; as above without the addition of glucose or maltose) or BMAA (basal/minimal media as prepared above), each without supplementation or supplemented with glucose, d-lactate, l-lactate or either lactate plus glucose. YCFA broth was prepared as described above with both glucose and maltose as carbon sources. For all media types, pH was measured prior to sterilization and adjusted to neutral conditions (pH=7), using drops of NaOH (4.5 N, Fisher SS256-500). All conditions were split across two 96-well plates to assess the growth of all six strains (CM02_02, CM02_05, CM03_47, CM06_29, CM06_28 and CM02_91A), assessed in triplicate across all six strains. All wells containing d- (Sigma #71716) or l-lactate (Thermo Fisher AAL1450014) had a final lactate concentration of 35 micromolar per well. Wells containing glucose (TCI G0048) had a final concentration of 1%. Each well, across all conditions, had a total volume of 200 µl. Negative control wells for each media type were also included to ensure sterility of the media. After preparation, the plate was sealed with a Breathe-Easy cover and placed into the Tecan Sunrise Microplate reader as described above.

Statistics on the growth curves from all platforms were generated in R using the package Growthcurver [71]. Heatmaps reflecting growth were created in R after area under the curve calculations (auc_l) using Growthcurver. Growth of bacterial strain directly corresponds to the value of log_10_ of area under the curve; that is, the higher the value, the more robust the growth in specific carbohydrate or d/l-lactate [72,73]. For the heatmap comparing *in vitro* vs. predicted substrate use in dbCAN3, PreBioM substrate names were matched to predicted sugar substrate names generated from CAZyme profiles (using the ‘run_dbCAN’ command), presuming ‘lentinan’ and ‘GOS/beta-glucan’ as beta-glucan; ‘chitosan’ as ‘chitin’; and ‘chondroitin sodium sulphate’ as ‘host glycan’ in PreBioM substrate names compared to dbCAN3 predicted sugar use, respectively.

### Data availability

All analyses were done through Clemson University’s high-performance computing center, Palmetto [74] and RStudio. All raw sequence data and associated information have been deposited in the NCBI Sequence Read Archive under BioProject PRJNA1241798. All code used to analyse data is available at https://github.com/SeekatzLab/Anaerostipes-genomes.

## Results

### *Anaerostipes* are prevalent members of the human gut microbiota

**Fig. 1.**
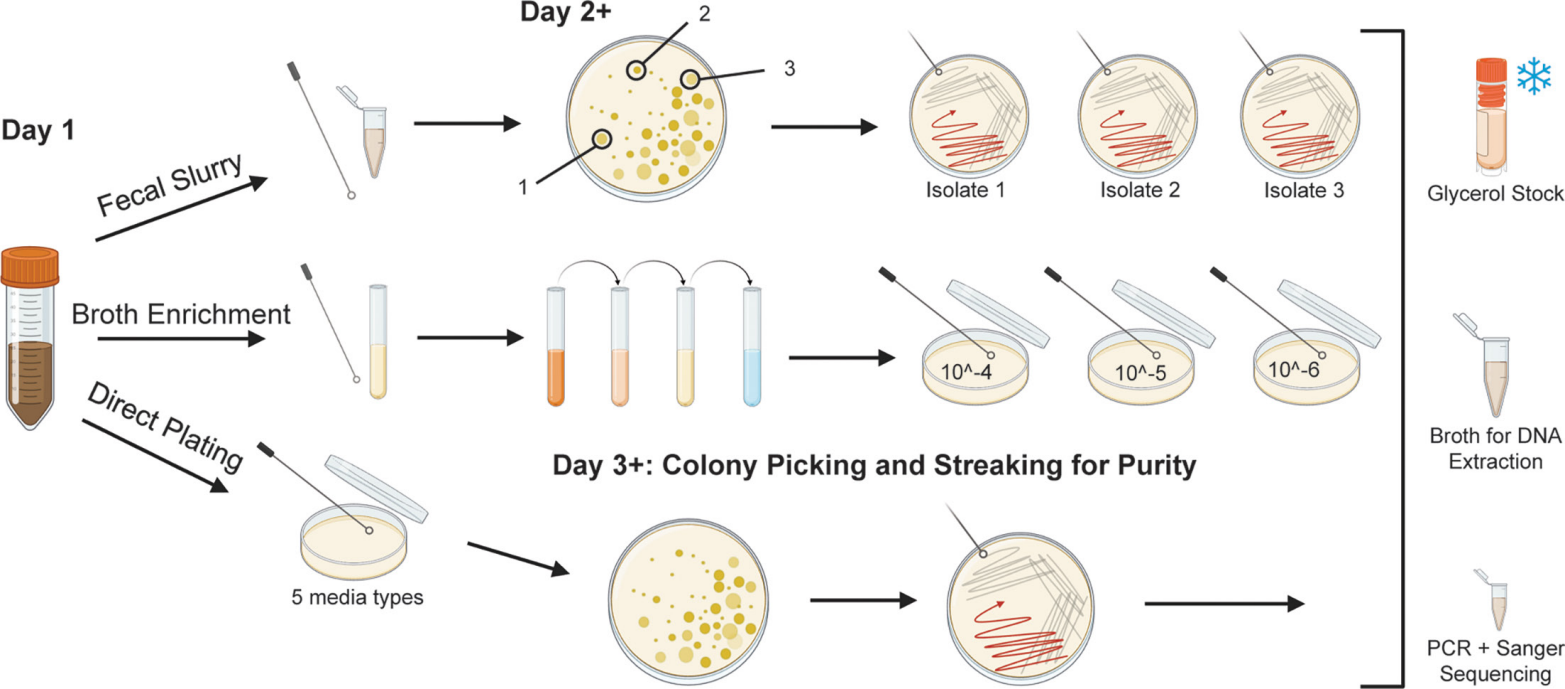
Isolation of human faecal bacteria. Graphical representation of the isolation method. Faecal samples (*n*=9) from healthy humans were either streaked directly, streaked as a slurry or enriched in liquid broth prior to dilution plating in different media types. Single, morphologically distinct colonies were streaked to purity and processed into glycerol stocks for long-term storage and DNA extraction for Sanger and whole genome sequencing. Created in BioRender. Seekatz, A. (2026) https:// BioRender.com/8xsf79y.

**Fig. 2.**
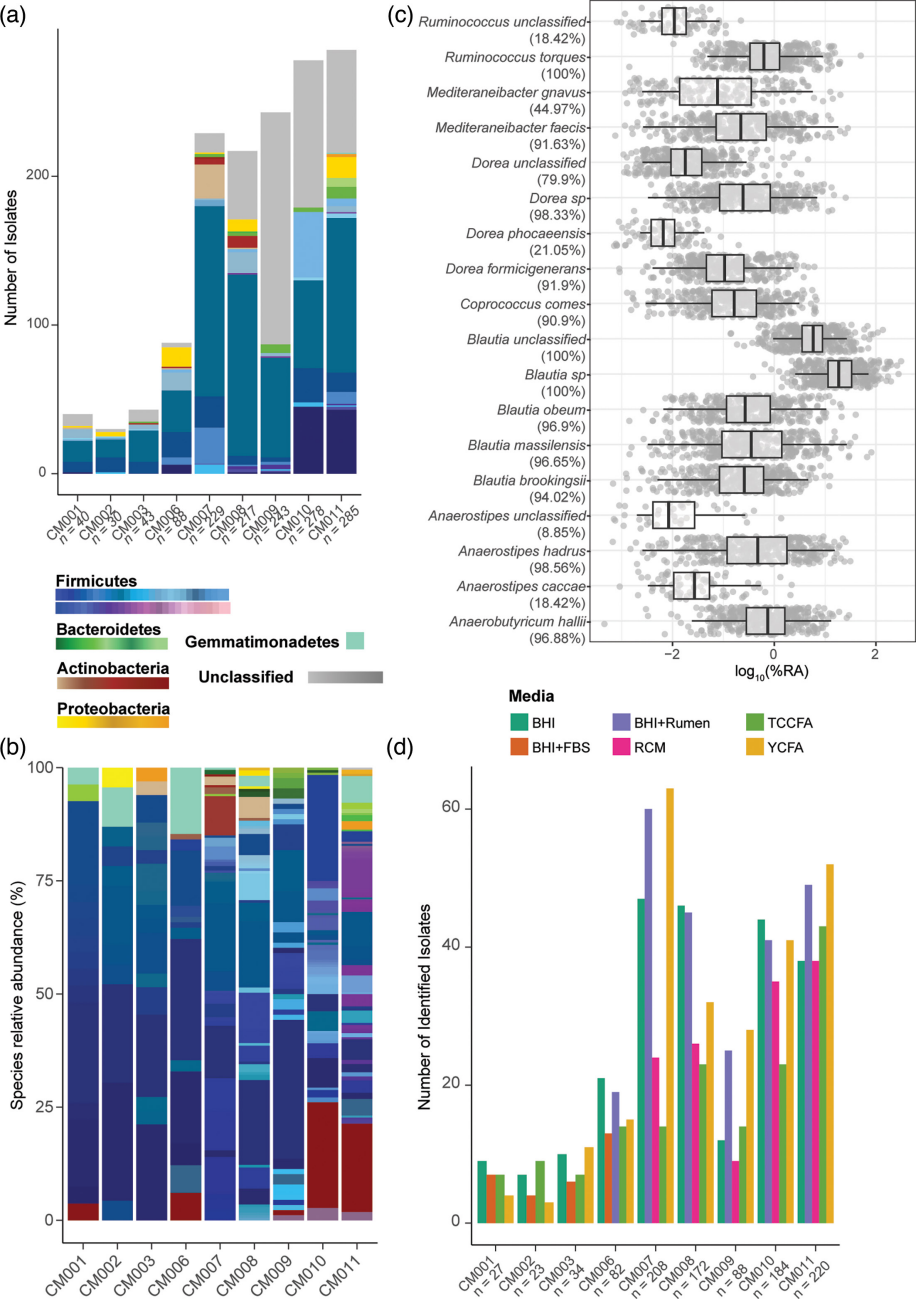
*Lachnospiraceae* are prevalent, abundant and frequently isolated from human faecal samples. (**a**) Total number of isolates obtained from the isolation protocol across nine human faecal samples, coloured by taxonomic classification at the phylum level (*n*=number of colonies picked during isolation). (**b**) Relative abundance of unique species identified per sample, colour-coded by genera. Legend for (a) and (b) represents phylum-level classification, with different genera shaded within each colour spectra. (**c**) Detection of select *Lachnospiraceae* species (using a custom classifier curated from isolated *Lachnospiraceae* species) in previously published 16S rRNA gene-based sequencing datasets from healthy human. Log_10_ of relative abundance of species is displayed on *x*-axis with prevalence (presence or absence of identified sequence). (**d**) Total number of isolates identified by Sanger sequencing, grouped and colour-coded by media type (*n*=number of colonies picked during isolation).

We screened nine fresh human faecal samples as part of a larger project aimed at the cultivation of various gut commensal bacteria (Fig. 1). Sanger sequencing of the full 16S rRNA gene from morphologically distinct colonies revealed a total of 1,038 isolates, representing 265 unique species. Of these, five phyla were identified, with representatives of the Firmicutes (88.05%), Proteobacteria (3.95%), Actinobacteria (3.85%), Bacteroidetes (2.79%) and Gemmatimonadetes (0.096%) (Fig. 2a, Table S3). Our method was effective at isolating members of the family *Lachnospiraceae*, with 542 total identified *Lachnospiraceae* isolates, representing the highest number of unique species (*n*=73, 27.55%) (Fig. 2b). Of these isolates, *A. hadrus* (*n*=139) was isolated multiple times across the nine total individuals, with at least one isolate retrieved from each individual, while *A. caccae* (*n*=11) was isolated from only three individuals (Fig. S1A). YCFA media retrieved the highest number of total isolates (*n*=249, 23.99%) and specifically *Lachnospiraceae* (*n*=149, 27.49%). However, BHI and BHI supplemented with rumen fluid (BHI+R) were also effective in targeting *Lachnospiraceae* species, with 139 isolates (25.65%) and 144 isolates (26.57%), respectively (Fig. 2d). Increasing the number of total colonies picked per sample also improved recovery of diverse species (Fig. S1B).

To broadly identify the prevalence of *Lachnospiraceae* species within the human gut, we created a custom classifier of the 16S rRNA gene from our sequenced isolates and mapped seven datasets of healthy adult faecal 16S rRNA amplicon sequences to these sequences (Fig. 2c). As observed previously [75], *Lachnospiraceae* species were highly prevalent, especially *Ruminococcus torques*, *A. hadrus*, *Blautia obeum*, *Blautia massilensis* and unclassified *Blautia* and *Dorea*, which were present in 95–100% of the samples from the datasets. Overall, relative abundance of most *Lachnospiraceae* species was low (~0–25%), except for unclassified *Blautia* and *Blautia* sp., which exhibited up to 70% relative abundance for some samples. While *A. hadrus* was detected in 98% of the samples, *A. caccae* was only detected in 19% of the samples, concurring with the proportions of these species identified from our isolation pipeline.

### *Anaerostipes* genus is defined by 12 unique species

**Fig. 3.**
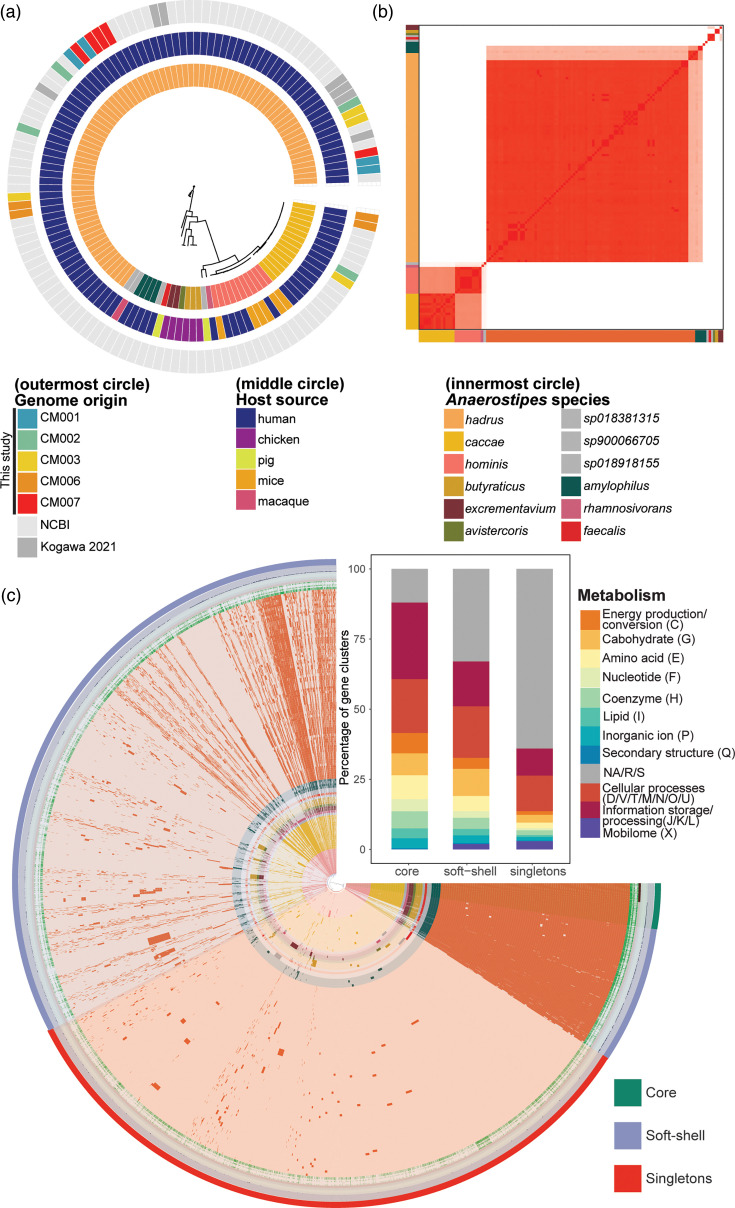
*Anaerostipes* genus is defined by 12 distinct species. (**a**) Maximum likelihood tree (unrooted) based on SNPs in the core genome across 126 *Anaerostipes* genomes, overlayed with species, host source and host subject (if isolated from this study) or published dataset. (**b**) ANI percentage across all 126 genomes (red denotes 100% ANI; white denotes 80% ANI). (**c**) Pangenome of *Anaerostipes* genus, as demonstrated by Anvi’o, displaying presence/absence of core (present in 100% of genomes), soft-shell (present in 99% of the genomes) and singletons (present only in a single genome across all genomes). Species colours on the *x* and *y* axes are designated in the legend. (Inset panel) Relative abundance of COG categories (colour in legend) representing core, soft-shell and singleton genes.

Given the prevalence of *A. caccae* and *A. hadrus* from our isolation pipeline, we sought to identify genomic diversity across the *Anaerostipes* genus. We compared whole genomes from a subset of our isolated *Anaerostipes* species (4 *A*. *caccae*; 17 *A*. *hadrus*) and 105 publicly available *Anaerostipes* genomes. An initial maximum likelihood tree (unrooted) based on SNPs demonstrated host-specific clustering at 90% bootstrapping, supported by bootstrapping 500 times (Figs 3a and S2A). Genomes classified as * A. hadrus*, *Anaerostipes amylophilus* and *A. caccae* were exclusively found in humans, whereas *Anaerostipes avistercoris*, * A. butyraticus* and *Anaerostipes excrementavium* were exclusively from chickens. Furthermore, multiple genomically divergent * A. caccae* or *A. hadrus* strains were isolated from the same human sample in our isolation pipeline, suggesting coexistence of strains. For instance, the faecal sample from subject CM003 yielded three *A. hadrus* and one *A. caccae* strains. An additional UPGMA tree built from AAI using EzAAI corroborated the SNP phylogeny (Fig. S2B).

To identify additional clades within and across species, we dereplicated and calculated the ANI using Anvi’o. This identified 12 species within the *Anaerostipes* genus at <95% ANI, with classification of these species matching their assigned taxonomy in GTDB (Fig. 3b), which was further validated at <95% AAI (Fig. S2C). Overall, the pangenome of the genus *Anaerostipes* was open (Fig. S3A), demonstrating a limited number of core genes (513 genes in all 126 genomes), with most genes categorized as soft-shell core genes (12,138 genes in at least 2 genomes) (Fig. 3c, Table S4). Most of the core genes within the genus were identified as related to amino acid synthesis and metabolism. Within amino acid production genes, most were specifically related to aromatic amino acid biosynthesis, including 3-dehydroquinate synthase, which catalyses the second step of Shikimate pathway [76]; Shikimate kinase, the fifth enzyme in the Shikimate pathway [77]; and aspartate aminotransferase, which catalyses the formation of oxaloacetate through the Krebs’ cycle [78] (Fig. 3c, inset panel). Most singleton genes (only present in one genome) were unknown (2,630 of 4,350 total singleton genes) as determined by the COG database. Of those identified, most genes belonged to transcription, defence mechanisms and cell wall biogenesis categories of COG.

### *Anaerostipes* genus can be divided into three functional clusters

**Fig. 4.**
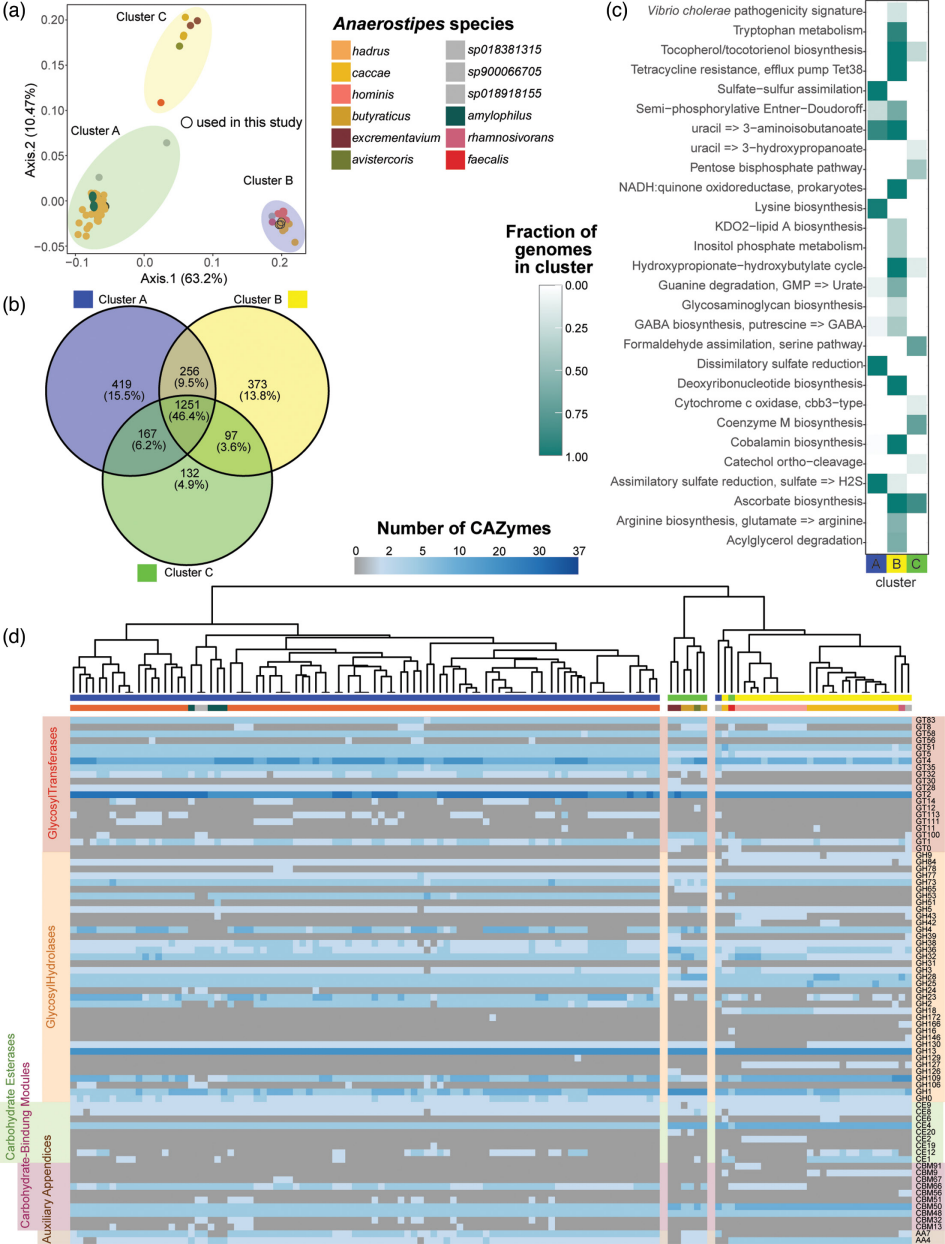
*Anaerostipes* clusters into three functional groups. (**a**) PCoA based on a Bray–Curtis distance matrix of presence/absence of gene assignments generated using Prokka, coloured by species. Clusters were assigned based on PAM. (**b**) Venn Diagram of predicted genes (Prokka) displaying total numbers and percentage of genes shared or unique to each cluster (*n*=2,696 total classified genes). (**c**) Differentially enriched KEGG modules across functional clusters (false discovery rate, *q*-value<0.05), coloured by fraction of genomes within each cluster. (**d**) Heatmap of number and type of CAZymes in individual genomes (*n*=126) using complete linkage clustering. GT=glycosyltransferase, GH=glycoside hydrolase, CE=carbohydrate esterase, CBM=carbohydrate-binding module, AA=Auxiliary appendices.

Functionally, the genomes split into three clusters, as determined by clustering using partitioning around medoids (PAM, average mean silhouette of the clusters=0.91) of the principal coordinates of axes analysis (PCoA) calculated from a Bray–Curtis dissimilarity index of gene predictions using Prokka (Fig. 4a). Cluster A (91 genomes) was dominated by *A. hadrus* genomes but also contained other human-associated genomes classified as *A. amylophilus*, *A. sp900066705* and *A. sp018918155*. Cluster B (28 genomes) was dominated by *A. caccae* and included the newly separated *Anaerostipes hominis* and *A. sp018381315* genomes, from either human or other mammalian host origin. Cluster C (7 genomes) was dominated by species isolated from chickens (*A. butyraticus*, *A. avistercoris* and *A. excrementavium*) and isolated from pigs (*Anaerostipes faecalis*). Given the host specificity of the three clusters, we sought to identify common and unique functional features among the three clusters. Almost half of the identified annotated genes (46.4% of 2,695 distinct genes total) were shared across all three clusters, with an average of 14 or 16% of genes unique to cluster A or B and 5% in cluster C (Fig. 4b). Clusters A and B shared more genes (9.5%) compared to clusters C and A (6.2%) or C and B (3.8%). Of the shared genes that were identifiable within the COG database, most genes were classified into functional category J: translation, ribosomal structure and biogenesis (14%), followed by E: amino acid transport and metabolism (10.7%) and G: carbohydrate transport and metabolism (9.9%). Within the 120 COG identifiable features exclusive to cluster A and 111 features exclusive to cluster B, E: amino acid transport and metabolism (10%) represented the largest functional category. However, for cluster C, which exhibited 33 exclusive features, G: carbohydrate transport and metabolism (15%) was the major functional category (Fig. S3D).

We also calculated functional enrichment across the clusters to identify differential representation of functional pathways (Fig. 4c). Diverse functional pathways were overrepresented in cluster B, including pathways associated with amino acid metabolism (tryptophan metabolism; arginine biosynthesis) and nucleotide metabolism (guanine ribonucleotide degradation), with all genomes possessing a putative cobalamin biosynthesis pathway. All genomes in cluster A exclusively possessed pathways for sulphate assimilation or reduction, lysine biosynthesis and pyrimidine degradation, whereas all genomes in cluster C exclusively possessed a coenzyme M biosynthesis pathway. However, module completion (defined as >75% complete) of many of the differentially identified pathways was only identified for lysine biosynthesis, one pyrimidine degradation pathway (uracil to 3-aminoisobutanoate) and sulphate assimilation (Fig. S4).

Variation of CAZymes was observed across the genomes, which closely matched clustering based on overall gene content above (Fig. 4d, Table S5). The glycosyltransferase category GT2 was the most numerous category of CAZymes observed across all genomes, although cluster A genomes demonstrated increased diversity of glycosyltransferases. Conversely, the diversity of carbohydrate esterases and carbohydrate-binding modules was higher in clusters B and C, suggesting an affinity to substrates different from cluster A, an indication of nutrient niches specific to the respective clusters.

### Distribution of genes associated with cobalamin and SCFA production varies across *Anaerostipes* functional clusters

**Fig. 5.**
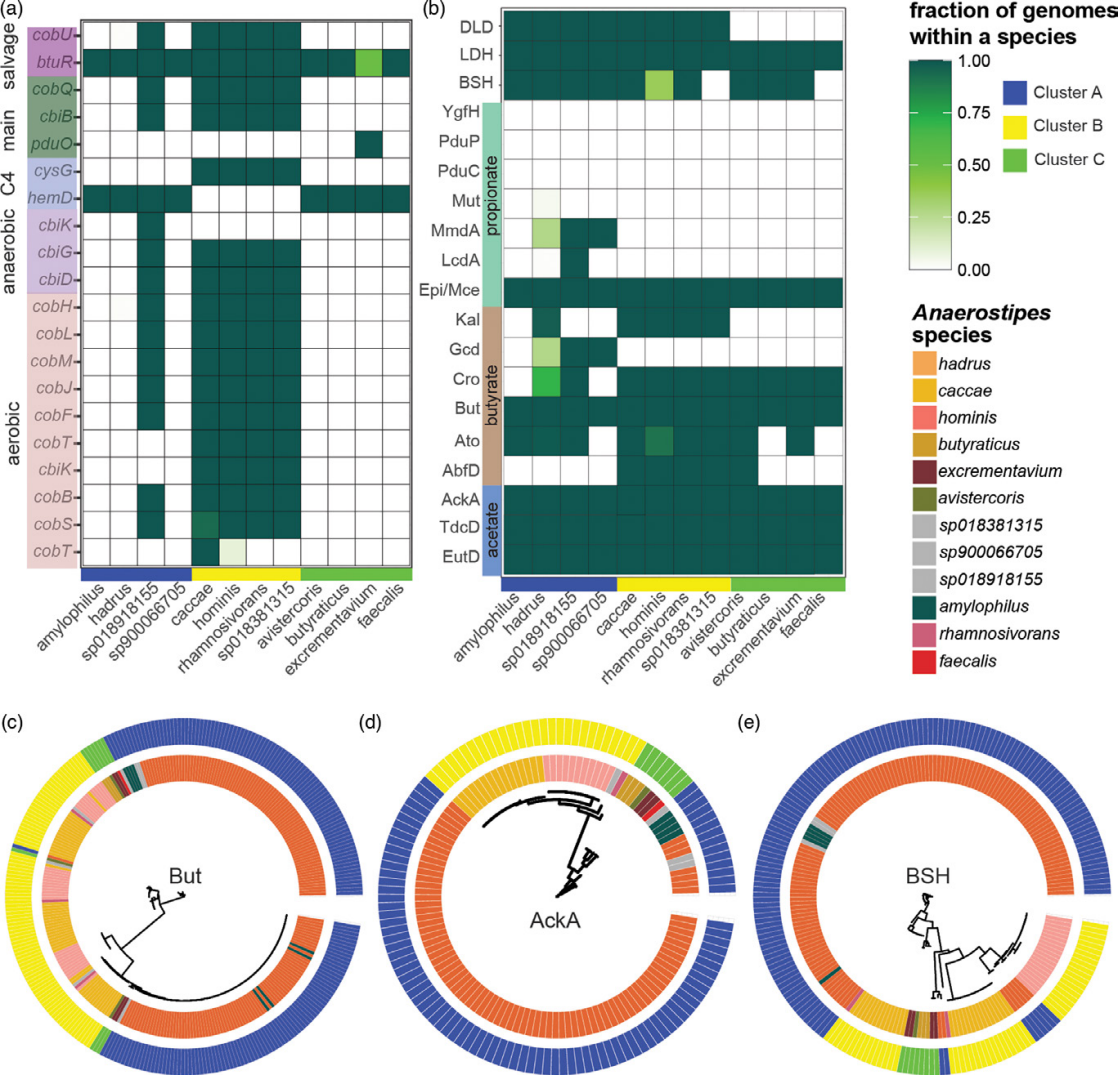
*Anaerostipes* species display varied capacity for cobalamin production, SCFA production and BSH. (**a**) Presence or absence of selected genes from aerobic, anaerobic, main [cob(II)yrinic acid a,c-diamide to adenosylcobalamin] salvage and C4 cobalamin production pathways represented as a fraction of genomes within a species, obtained from COG database using Anvi’o. (**b**) Presence or absence of select protein sequences from acetate, butyrate and propionate pathways, LDH, DLD and BSH, represented as a fraction of genomes within each species. (**c**) Maximum likelihood tree (unrooted) generated from butyryl-CoA:acetate CoA-transferase (But) protein hits (identity>60%) in all genomes (bootstrap *n*=100). (**d**) Maximum likelihood tree (unrooted) generated from acetate kinase (AckA) protein hits (identity>60%) in all genomes (bootstrap *n*=100). (**e**) Maximum likelihood tree (unrooted) generated from BSH protein hits (identity>60%) in the genomes (bootstrap *n*=100).

We next sought to identify how genes previously associated with select *Anaerostipes* species were distributed across all genomes. Corrinoids, such as cobalamin or B12, are important for gut microbial physiology since they are an essential co-factor for corrinoid-dependent enzymes [79]. Production of cobalamin by *A. caccae* has been postulated, as co-cultures of *Akkermansia muciniphila* and *A. caccae* have been demonstrated to produce low propionate levels through corrinoid-dependent methylmalonyl-CoA mutase enzymes in *Ak. muciniphila* [80]. We observed that very few species in cluster A (containing *A. caccae*) possessed genes belonging to the main, aerobic, anaerobic, C4 and salvage pathways to produce cobalamin (Fig. 5a). While only the salvage pathway, represented by *cobU* and *btuR*, was present in cluster B genomes, both the main and aerobic pathways were complete or near complete, only missing the presence of the final genes *pduO* (adenosyltransferase) and *cobST* (cobalamin-5′-phosphate synthase) in some strains. This suggests modified corrinoid production or a yet novel aspect of cobalamin production as hypothesized previously [80,81]. The anaerobic pathway in most genomes lacked *cbiK* (anaerobic cobalamin biosynthetic cobalt chelatase), which may corroborate previous co-culture observations requiring the presence of additional bacteria to produce cobalamin [80]. In contrast, clusters A and C lacked almost all cobalamin-related genes, with the exception of the *sp018918155* genome in cluster A.

A major health-related function attributed to *Anaerostipes* is the production of SCFAs, especially butyrate [23]. Comparing our Prokka predicted proteins from the whole genomes to specific UniProt protein sequences, we observed widespread presence of butyrate- and acetate-related proteins across all species and functional clusters, with some variability associated with the functional clusters (Fig. 5b). Lactate metabolism via both NAD-dependent l-lactate dehydrogenase (LDH) and NAD-independent d-lactate dehydrogenase (DLD) [82,83] was observed in all species, with the exception of DLD, which was absent in cluster C genomes. Importantly, the main butyrate-associated terminal enzyme in butyrate production, But (butyryl-CoA:acetate CoA-transferase), was present in all genomes. Although *Anaerostipes* is not known to display Buk (butyrate kinase) activity [31,84, 85], our preliminary search for Buk protein sequences yielded a 64.9% identity match to AckA (acetate kinase) at 100%. An unrooted maximum likelihood tree of the aligned amino acid sequences for But and AckA demonstrated species-specific clustering with low bootstrap values (<50%, *n*=500), suggesting near identical sequences across all genomes (Fig. 5c, d). The presence of other SCFA genes was more specific to functional clusters. Most cluster B and C genomes lacked Gcd (glutaconyl-CoA decarboxylase), which catalyses conversion of glutaconyl-CoA to crotonyl-CoA via the glutarate pathway of butyrate biosynthesis, and cluster C genomes lacked Kal (3-aminobutyryl-CoA ammonia lyase), which catalyses conversion of 3-aminobutyryl-CoA to crotonyl-CoA. In contrast, only Epi/Mce (methylmalonyl-CoA epimerase), which converts succinate to propionate, was present across all genomes [86]. Some cluster A genomes exhibited the presence of MmdA (methylmalonyl-CoA decarboxylase), which catalyses *S*-methylmalonyl-CoA to propionyl-CoA, and LcdA (lactoyl-CoA dehydratase), which converts lactoyl-CoA to acryloyl-CoA [87].

An important function of some commensal gut microbes is deconjugation of primary BAs, performed through bile salt hydrolases (BSH). All species demonstrated the presence of BSH, with the exception of *A. faecalis* and *sp018381315*, which were only represented by one genome each, and a few *A. hominis* genomes (Fig. 5b). Although bootstrap values for amino acid sequence comparison represented by BSH were low, some sequences were more closely related to each other than others, such as AGGJHBAO_01892 from CM03_47 (*A. hadrus*), OMENAAMB_02178 from CM02_14 (*A. hadrus*) and EGPMHLCL_02247 from CM03_84 (*A. hadrus*), for which bootstrapping values were at 100%, even though all three strains were isolated from two different human subjects (Fig. 5e).

### Pangenomic comparisons of *A. caccae*, *A. hominis* and *A. hadrus* demonstrate functional differences

**Fig. 6.**
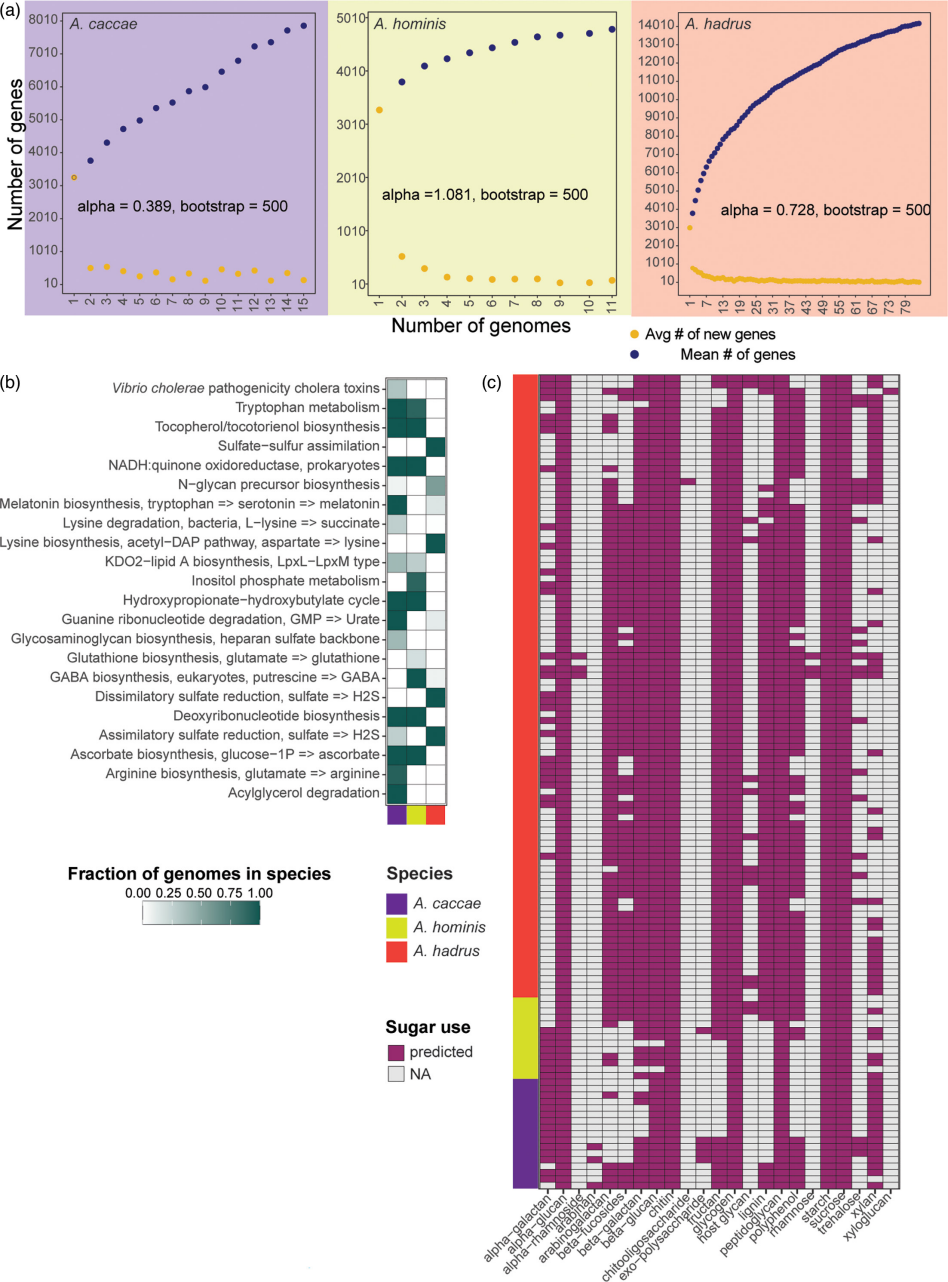
Human-associated *Anaerostipes* species, *A. hadrus*, *A. caccae* and *A. hominis*, vary in functional capacities. (**a**) Number of genes as a function of number of genomes depicting number of new genes in yellow dots and number of genes in the pangenome in blue dots of *A. caccae* (*n*=15, purple), *A. hominis* (*n*=11, seagreen) and *A. hadrus* (*n*=84, red). Alpha equals the power law estimate, run over 500 iterations using micropan in R (*P* value<2e^−16). (**b**) Differentially enriched KEGG modules across the three species (false discovery rate, *q*-value<0.05), coloured by fraction of genomes. (**c**) Presence (purple box) or absence (grey box) of sugar substrate (*x*-axis) predicted from CAZymes across all the genomes in the three species.

We next focused on comparing functional differences within and across three prevalent human-associated *Anaerostipes* species. Pangenomic comparisons within *A. hadrus* (84 genomes), *A. caccae* (15 genomes) and *A. hominis* (11 genomes) species suggested that both *A. hadrus* and *A. caccae* displayed open pangenomes (alpha<1), whereas *A. hominis* demonstrated a closed pangenome (alpha>1) (Fig. 6a). Based on identified COG genes, *A. hadrus* demonstrated the smallest core genome among the three species, with conservation of J: translation, ribosomal structure and biogenesis (representing 10.69%) across the *A. hadrus* core genome, while the maximum genes belonged to G: carbohydrate transport and metabolism genes (8.75%) and X: mobilome genes (3.9%) in soft-shell and singleton categories, respectively (Fig. S5). This was in comparison to *A. caccae* and *A. hominis* core genes, where G: carbohydrate transport and metabolism comprised 12.7 and 13.1% of core genes, respectively. *A caccae* and *A. hominis* also exhibited a smaller percentage of X: mobilome genes (0.14 and 0.61%, respectively) (Fig. S5).

To investigate functional differences across the three species, we used Anvi’o to identify differentially enriched modules. *A. caccae* and *A. hominis* shared more similarities compared to *A. hadrus*, including exhibiting pathways associated with tryptophan metabolism, tocopherol/tocotrienol biosynthesis and NADH:quinone oxidoreductase (Fig. 6b). Melatonin and arginine biosynthesis, as well as guanine ribonucleotide and acylglycerol degradation, were almost exclusively observed in *A. caccae*, whereas *A. hominis* genomes exhibited inositol phosphate metabolism and GABA biosynthesis pathways. In comparison, *A. hadrus* demonstrated widespread presence of multiple pathways associated with sulphate reduction and lysine biosynthesis.

We also used dbCAN3 to predict the substrate carbohydrate use across all genomes (Fig. 6c). Several substrate predictions were conserved across all three species, including alpha-glucan, starch, sucrose-6-phosphate and sucrose, despite genomic variation observed within the species. Arabinan, alpha-glucan, xyloglucan and exopolysaccharides were not predicted to be metabolized by these three species. Use of lignin, beta-glucan, polyphenol and beta-fucosides seemed restricted to *A. hadrus*, and *A. caccae* genomes generally lacked CAZymes involved in fructan, inulin and polyphenol utilization, suggesting that *A. caccae* may exhibit a more restricted carbohydrate use profile. Comparison of individual CAZymes followed this pattern, whereby *A. caccae* demonstrated a less diverse CAZyme profile (Fig. 4d).

### *Anaerostipes* species exhibit variation in factors associated with colonization

While 16S rRNA gene-based studies commonly associate *Anaerostipes* with health, some studies have identified associations with particular disease states [88,89]. We screened all *A. caccae*, *A. hadrus* and *A. hominis* genomes against virulence and antibiotic resistance gene databases using PathoFact [68]. All genomes demonstrated the presence of toxin-antitoxin systems, which may represent conserved plasmid and transposon maintenance (Fig. S6). All *A. caccae* and *A. hominis* genomes exhibited the presence of exfoliative toxins A and B, initially identified on *Staphylococcus aureus* [90]. Some strains of *A. caccae* also exhibited the presence of a gene encoding a zona occludens toxin, an enterotoxin located on prophages of some pathogenic *Vibrio* species [91]. Most * A. hadrus* and *A. hominis* genomes also exhibited the presence of a gene encoding an insecticidal toxin complex protein, TccC, that has been associated with increased colonization ability of some pathogenic *Yersinia* strains [92,93].

**Fig. 7.**
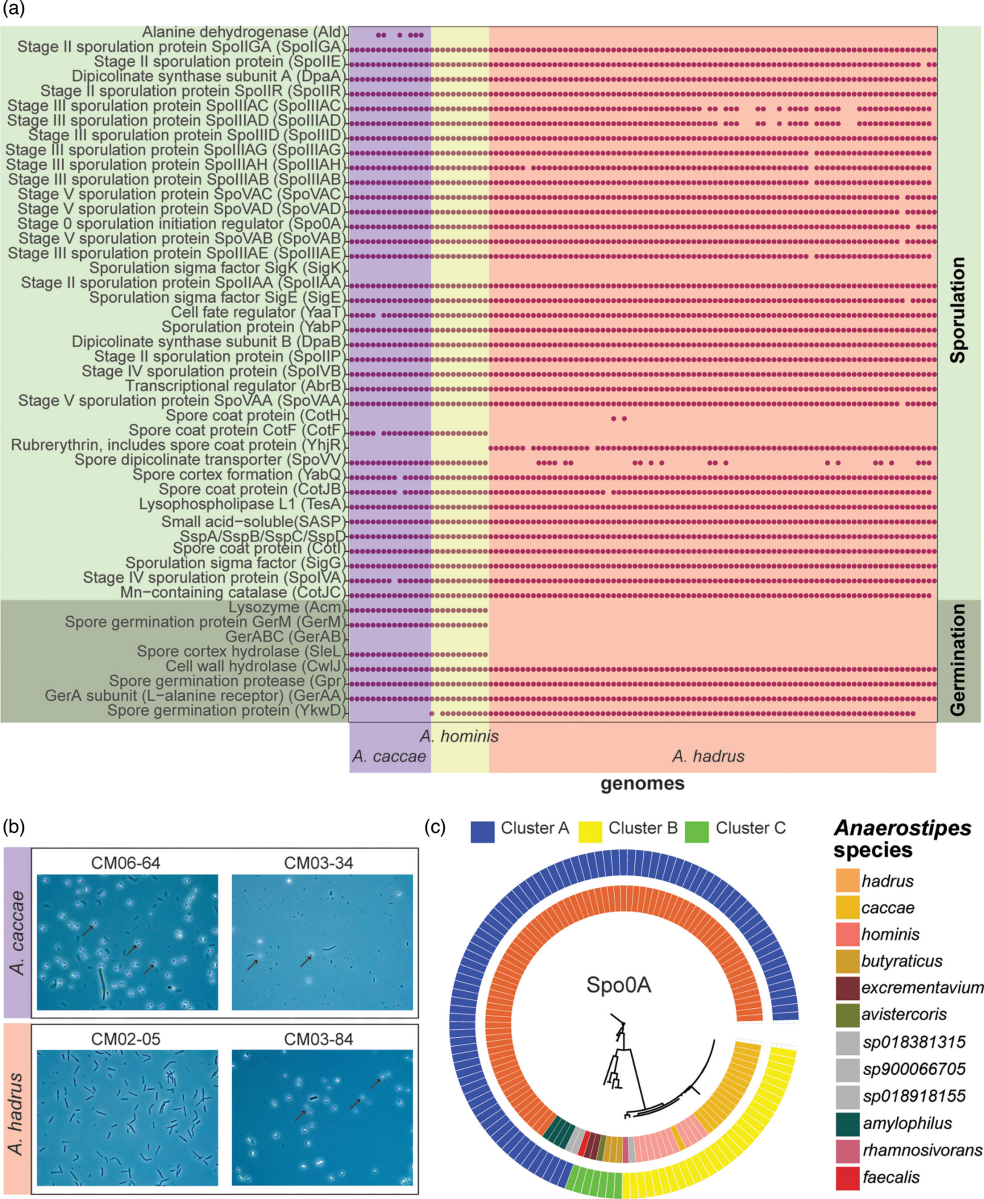
*A. caccae* and *A. hadrus* isolates display common antibiotic and sporulation genes. (**a**) Presence (purple dot) or absence (no dot) of antibiotic resistance, sporulation and germination genes across *A. caccae* (*n*=15), *A. hominis* (*n*=11) and *A. hadrus* (*n*=84) genomes. Antibiotic resistance was detected through PathoFact and sporulation or germination genes through KOfam. (**b**) Phase contrast of spores from two *A. caccae* and two *A. hadrus* strains isolated in this study. Spores marked by arrows (no spores detected in CM02_05 strain). (**c**) Maximum likelihood tree (unrooted) for Spo0A protein hits (identity>60%) across all 126 genomes (bootstrap *n*=100).

We also used PathoFact to investigate the putative antimicrobial profiles across the three major *Anaerostipes* species. All genomes contained widespread glycopeptide, macrolides, lincosamides and streptogramines and efflux pump encoding multidrug resistance genes, which can be attributed to either accumulation of mutations on exposure or dissemination of resistance through horizontal gene transfer across the gut microbial community [94] (Fig. 7a). *A. hominis* genomes and some strains of *A. hadrus* exclusively exhibited genes encoding beta-lactam and polymyxin resistance, which were absent in all *A. caccae* genomes. Genes associated with resistance to other antibiotics, such as tetracycline, aminoglycosides and bacitracin, were observed sporadically across the three species.

Finally, we were interested in identifying potential sporulation and germination pathways in *A. hadrus*, *A. hominis* and *A. caccae* genomes. The formation of endospores across various *Anaerostipes* species has been observed but not fully characterized [21]. All three species exhibited the presence of most genes required to sporulate through the mechanism characterized for *Bacillus subtilis* [95], including *spo0A* (sporulation initiation protein), stage II, stage III and stage V sporulation and various spore coat genes (Fig. 7a). Some species-specific conservation was observed, such as the presence of *ald* (alanine dehydrogenase) exclusively in *A. caccae* genomes and *yhjR* (rubrerythrin, spore coat) exclusively in *A. hadrus*. Given the previously observed variability in *Anaerostipes* spore formation [21], we tested the ability of two *A. caccae* and two *A. hadrus* strains to form spores *in vitro*. Both * A. caccae* strains formed visible spores after 14 days of growth in Clospore media, while only one *A. hadrus* strain produced visible spores under phase contrast microscopy (Fig. 7b).

We observed limited variation within sporulation and germination genes between *A. hadrus* and *A. caccae* strains. However, on closer inspection of the genomes, we found that the two tested *A. caccae* strains, CM03_34 and CM06_64, did not have *ykwD* or *yhjR*, two uncharacterized predicted spore-related genes previously identified in *B. subtilis*, whereas *A. hadrus* strains did not possess genes for *cotF* (spore coat protein), *acm* (lysozyme M1; 1,4-beta-*N*-acetylmuramidase), *sleL* (spore cortex hydrolase), *gerM* (spore germination protein) or *spoVV* (spore dipicolinate transporter) based on the COG database. In addition to these species-level differences, the two tested *A. hadrus* strains exhibited some genomic differences in sporulation and germination genes, which may explain their different sporulation outcomes. These included differences across the sporulation genes *spoIVFB*, *spoIIP*, *yabP*, *spoVAD* and *spoIIID* and CM02_05, for which we did not observe sporulation with our methods, did not seem to possess *spoVAC* at all, an important germination-related mechanosensitive channel in *B. subtilis* [96].

Given the conservation of *gerA* across these species, we further sought to compare *gerAA*, *gerAB* and *gerAC* (the three genes encoding each of the three proteins involved in a functional GerA receptor) [97] from COG profiles. Comparison to the COG database identified only one *gerA* protein, GerAA, in all three genomes, but no GerAB in any genomes. In contrast, use of a UniProt database using DIAMOND identified the presence of *gerAB* homologues, which encodes for the protein that typically binds to the germinant signal, but no *gerAA*, which encodes for the protein that transduces a downstream signal [97], suggesting a discrepancy in database identification. The *gerAC* gene, which encodes for a lipoprotein with an unknown function [98], was not detected in any species. Aligning the GerAB protein sequence using DIAMOND revealed tight clustering by species (bootstrapping>50%), suggesting species-specific germination pathways (Fig. 7c).

### Predicted and actualized sugar use varies across *Anaerostipes* strains

**Fig. 8.**
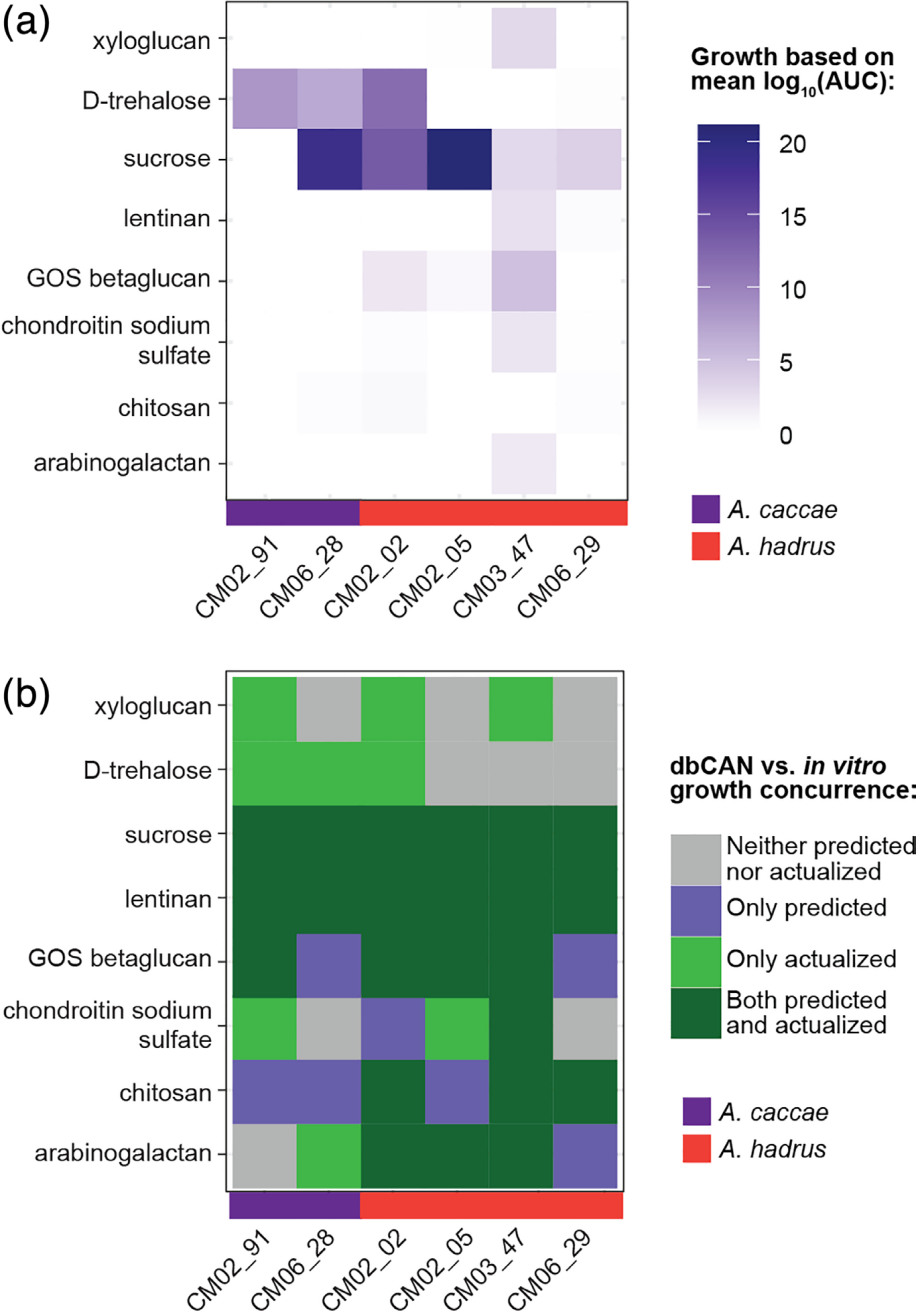
*A. hadrus* and *A. caccae* strains demonstrate strain-dependent growth in defined carbohydrate sources. (**a**) Heatmap of *A. hadrus* and *A. caccae* strain growth in selected minimal media (BMAA) supplemented with select carbohydrates using Biolog PreBioM plates, represented by log_10_(area under the curve) averaged over triplicates per strain. (**b**) Heatmap of concurrence between predicted (per dbCAN3 results) and actualized growth *in vitro* (dbCAN3 matched names in parentheses).

To examine differences in carbohydrate use across strains, we selected six representative strains (two *A. caccae* species and four *A. hadrus* species, selected from a 99% dereplication cutoff across genomes) to examine their ability to use distinct carbohydrate sources *in vitro*. Of the eight sugars that matched between dbCAN3 predicted use and our *in vitro* platform (i.e. Biolog PreBioM plates using our defined BMAA minimal media), only sucrose supported growth across most strains when supplemented as a single carbon source in minimal media (Figs 8a and S7). Growth in other matched supplemented sugars was variable across the strains, although some species-specific patterns were observed, such as chitosan supporting growth of one *A. hadrus* strain (CM02_02) and d-trehalose supporting growth of both *A. caccae* strains and one *A. hadrus* strain. When *in vitro* growth was compared to the best-matched dbCAN3 predicted substrates, additional mismatches and strain variability were observed. For instance, strain CM03_47 (*A. hadrus*) displayed matched actualized and predicted growth across most matched sugar conditions, with the exception of xyloglucan (actualized) and chitosan (‘chitin’, predicted via dbCAN3). In contrast, CM06_29 (*A. hadrus*) was predicted to grow on multiple sugar sources but was never actualized *in vitro*, and growth on trehalose or xyloglucan was not genomically predicted for any strains but was observed *in vitro*.

Previous studies have demonstrated the ability of *Anaerostipes* species to produce butyrate from lactate [23,24, 31, 83, 99], also predicted by the presence of LDH and DLD in our study (Fig. 5). Compared to glucose-supplemented minimal media (BMAA) or bYCFA, limited growth as measured by OD_600_ was observed when either basal media was supplemented with d-lactate or l-lactate as a single carbon source (Figs S7 and S8). Strains CM03_47, CM02_02 (*A. hadrus*) and CM06_28 (*A. caccae*) demonstrated growth in bYCFA supplemented with d-lactate, whereas strains CM03_47 and CM02_05 (*A. hadrus*) grew in bYCFA supplemented with l-lactate. However, unsupplemented bYCFA alone also supported the growth of many strains. The addition of glucose alongside either lactate supported growth of all strains. Notably, we observed considerable variability in growth across the strains under each substrate condition.

## Discussion

Our study aimed to genomically characterize species within *Anaerostipes*, a genus known to inhabit the human gut microbiome. *Anaerostipes* species have been identified across global human populations [85] and other animal hosts [100]. Using both cultivation and 16S rRNA comparisons, we observed high prevalence of *Anaerostipes* species in the human gut, particularly for the species *A. hadrus*. Using genomic comparisons, *Anaerostipes* genomes could be divided into three main functional clusters that correlated with host origin and demonstrated genomic capabilities (i.e. vitamin B12 production) in addition to widespread genes involved in SCFA production. Importantly, for prevalent human-associated *Anaerostipes* species (*A. hadrus*, *A. caccae*, *A. hominis*), we observed both genomic and phenotypic strain-based variability in carbohydrate use. These data, alongside multiple studies correlating *Anaerostipes* with health [88,89, 101, 102], support a commensal (and thus a potentially important) role of *Anaerostipes* in the human gut [103].

Isolation of multiple *Anaerostipes* species revealed that multiple ‘strains’ (defined as phylogenetic clusters with a 99% ANI within a species in our study) were present in a single individual. For example, a faecal sample from subject CM003 yielded three * A. hadrus* strains and one *A. caccae* strain. This suggests coexistence between different strains of the same species within the same host and the existence of strains of different species of *Anaerostipes* within the same host. Similar results have been reported for *Escherichia coli*, *Ruminococcus gnavus* and *Bacteroides vulgatus*, where multiple strains of the same bacteria have been identified within the same individual [104]. We also observed the existence of two highly similar strains of *A. caccae* (dereplicating at 99% ANI) across different individuals (CM002 and CM003), demonstrating that specific *Anaerostipes* species may be generalizable across the human gut.

Our phylogenetic, AAI and ANI comparisons suggest high intra-species similarity within the 12 *Anaerostipes* identified in our genomic analyses. Although some unnamed species were represented by single genomes (thus requiring further validation), these species phylogenetically aligned with host specificity, with species such as *A. avistercoris* and *A. excrementavium* originating exclusively from chickens and *A. hadrus* and *A. caccae* originating from humans. This suggests adaptation of particular *Anaerostipes* species to their respective host environment, similar to co-speciation observations made for other gut symbionts such as *Bifidobacterium* and *Enterococcus* observed across host environments [105,106]. A previous study compared 527 assembled genomes from isolates and metagenome-assembled genomes and found three main phylogenetic clusters within *A. hadrus* without association across geographical regions [85]. Our study, which included 41 genomes from this study that passed our quality control measures, did not observe large-scale functional clustering within *A. hadrus*, although we did observe 39 fairly homologous clusters at 99% ANI (obtained from Anvi’o). Inclusion of additional high-quality *Anaerostipes* genomes with increased global representation might distinguish additional phylogenetic clusters that could be better associated geographically or to a specific health context.

Across the *Anaerostipes* genus, we observed three main clusters that also correlated with host source. While cluster A (*A. hadrus*-dominated) was almost exclusively described by human-sourced isolates, cluster C included genomes sourced from chickens and pigs, whereas cluster B consisted of genomes sourced from humans, mice and pigs. The impact of host physiology and phylogeny on the gut microbiome has been characterized in non-human primates, wherein specific gut microbiome signatures have been associated with host species despite similar dietary niches of the hosts [107]. Convergent evolution of gut bacterial species to perform similar functions in different hosts, while typically overlooked if only assessing taxonomy or phylogenetic similarity, has been observed across other hosts, such as within speciation of *Enterobacteriaceae* species across different *Caenorhabditis* species, *Caenorhabditis elegans* and *Caenorhabditis briggsae* [108].

Both shared and differentiated features across the three identified functional categories further suggested independent roles or adaptation for *Anaerostipes* species in the gut. All species exhibited the canonical But (butyryl-CoA:acetate CoA-transferase) protein that has defined *Anaerostipes* as a butyrate-producing genus. Additionally, all species exhibited the presence of AckA (acetate kinase), TdcD (acetate propionate kinase) and EutD (phosphotransacetylase), associated with acetate production [109], and Epi/Mce (methylmalonyl-CoA epimerase), associated with propionate production [110]. Almost all species exhibited the presence of BSH, adding to the growing list of bacteria capable of modifying host-produced primary BAs [111]. However, differences across the functional clusters, which also displayed host specificity, may further distinguish species niches within the gut. Cluster B genomes (*A. caccae*-dominated) exhibited pathways for tryptophan metabolism, as well as other biosynthetic pathways, to produce arginine, tocopherol (vitamin E) and cobalamin (vitamin B12). A closer comparison of cobalamin production revealed that *A. caccae* and other functionally similar species exhibited multiple cobalamin biosynthetic pathways, which have not been previously described in *Anaerostipes* specifically [112,113]. Although actual cobalamin production would have to be experimentally validated, the near completeness of these pathways suggests that *A. caccae* may play at least a cross-feeding role in cobalamin production in the gut [80]. Species within cluster A exhibited more diverse butyrate production pathways compared to cluster B or C, although clusters A (*A. hadrus*-dominated) and B (*A. caccae*-dominated) also exhibited Kal (3-aminobutyryl-CoA ammonia lyase) and Cro (crotonase/enoyl-CoA hydratase). Comparison of But, AckA and BSH protein sequences across all species indicated conserved protein sequences through low bootstrap/branch support values.

Further genomic investigation of the three most dominant human *Anaerostipes* species (*A. hadrus*, *A. caccae* and *A. hominis*) revealed additional information relevant to human health. Both *A. caccae* and *A. hadrus* exhibited open pangenomes, a feature of sympatric bacteria living in close ecological contact with each other and suggesting additional gene diversification is possible should larger-scale comparisons within the species be conducted [114]. In contrast, *A. hominis* exhibited a closed pangenome, a characteristic of an allopatric lifestyle, where organisms living in isolated environments have limited access to external genetic materials [115,116]; however, the number of *A. hominis* genomes in our analysis was low (*n*=11), and additional genomes to its pangenome might reveal more novel genes, particularly given the presence of mobilome genes in the singleton pangenome bin. In terms of predicted antibiotic resistance genes, *A. hadrus*, *A. caccae* and *A. hominis* displayed some species-specific differences, with *A. caccae* lacking predicted beta-lactam and polymyxin resistance genes. Both *A. caccae* and *A. hominis* displayed the presence of more putative virulence genes compared to *A. hadrus*, including homologues for a *Vibrio* zona occludens toxin [117] and *Staphylococcus* exfoliative toxins [118]. Despite some studies associating *A. caccae* with disease states rather than health [119], there is no functional evidence of toxins in *Anaerostipes*, a limitation of our genomic study. Nevertheless, these toxin genes could be an artefact of horizontal gene transfer within gut commensals, where virulence genes are indiscriminately acquired by gut bacteria but do not lead to pathogenesis [120], as in the case of *Neisseria* [121], a gut commensal known to exchange virulence genes between pathogenic and non-pathogenic strains.

Understanding sporulation and germination mechanisms of gut anaerobes is perhaps most relevant to the development of microbial therapeutics, given that spore delivery is an optimal method to deliver anaerobic bacteria to the gut [122]. Although spore formation is encompassed by many commensal *Clostridia* and specifically *Lachnospiraceae* [123], sporulation and germination mechanisms are incompletely described for most species other than *B. subtilis* and the gut pathogen, *C. difficile* [124,126]. All three major human *Anaerostipes* species possessed sporulation- and germination-associated genes observed in *B. subtilis*, which sporulates via Spo0A phosphorylation and germinates through Ger proteins with GerAB that bind to the germinant nutrient l-alanine [124,125]. Initial comparisons using the COG database identified GerAA in the three *Anaerostipes*; however, closer phylogenetic analysis revealed that this was likely GerAB instead. We also observed a species-specific clustering of GerAB protein sequence informed by the bootstrapped maximum likelihood tree. This suggests *Anaerostipes* species might follow a germination mechanism similar to *B. subtilis*. While a previous study by Kadowaki *et al.* was not able to identify *in vitro* production of spores despite the identification of similar spore-related genes in *A. hadrus* [21], we observed *in vitro* spore production of both tested *A. caccae* strains and one *A. hadrus* strain. We also observed the absence of some sporulation genes, such as *spoII*, in CM02_05, the strain that did not produce spores. This suggests possible strain-to-strain variation across *A. hadrus* sporulation, warranting additional validation and comparison using lab-based methods.

Our study aimed to genomically characterize *Anaerostipes* species and is thus limited to genomic predictions and functional potential. Results from our *in vitro* growth of a subset of strains on carbohydrates that matched some of our substrate predictions (from dbCAN3) demonstrated that less than half of the genome-encoded predictions were actualized, i.e. the strains could grow in minimal media (BMAA) with a single carbohydrate as a carbon source. We have observed similar mismatches in genome-to-phenotype comparisons previously, notably in phylogenetically similar strains isolated from the same individual, demonstrating potential limitations of genomic predictions based on databases alone [127]. Other caveats of our study include different experimental conditions (i.e. methods to measure growth vs. substrate use; media) that may obscure comparisons across studies. Previous studies have demonstrated lactate consumption by *Anaerostipes* species when supplemented with lactate as a sole carbon source [83]. We did not measure consumption or production of specific products (i.e. using methods such as gas chromatography-mass spectrometry) and thus cannot conclude consumption of lactate in our experiments. However, our results did demonstrate variable, if limited, growth across strains in the presence of d- or l-lactate under different media conditions, indicative of intra-species variation. While these results certainly highlight the importance of validating genomic-based analyses and potential limitations in current genomic tools, they also suggest additional mechanisms beyond phylogenetic similarity for phenotypic diversification within closely related species. This might be especially true for more restricted environments such as the gut, where intra-species diversification has been previously observed [128]. In the context of human health, diversification of nutrient use within a species might aid niche partitioning to uphold community-level diversity, which is known to increase stability to potential perturbations [129]. Further comprehensive validation of genome-encoded functions across larger isolate collections is necessary for additional insights, particularly in the context of strain variation.

Overall, our study aimed to define genomic diversity across *Anaerostipes* species, elucidating genomic strain diversity that might influence previous associations based on 16S rRNA gene-based analyses alone. Our data suggest that while *Anaerostipes* species might be host-restricted, strain-level differences across and within *Anaerostipes* species may explain their adaptation to particular host gut niches.

## Supplementary material

10.1099/mgen.0.001617 Uncited Supplementary Material 1.

10.1099/mgen.0.001617 Uncited Supplementary Material 2.

## Abbreviations

AAI: average amino acid identity
ANI: average nucleotide identity
BAs: bile acids
BHI: brain heart infusion
BMAA: basal media with amino acids
BSH: bile salt hydrolases
bYCFA: basal YCFA
CAZymes: carbohydrate-active enzymes
COG: Clusters of Orthologous Groups
DLD: d-lactate dehydrogenase
GC: gas chromatography-mass spectrometry
GTDB: Genome Taxonomy Database
IBS: irritable bowel syndrome
KEGG: Kyoto Encyclopedia of Genes and Genomes
LDH: l-lactate dehydrogenase
PAM: partitioning around medoids
PCoA: principal coordinates analysis
SCFAs: short-chain fatty acids
UPGMA: unweighted pair group method with arithmetic mean
YCFA: yeast casitone fatty acids
