# Supplementary material for: Genomic insights into the functional and metabolic versatility of gut microbiome Anaerostipes species
Source: Microb Genom. 2026 Feb 9;12(2):001617. doi: 10.1099/mgen.0.001617 (PMC12888040; doi:10.1099/mgen.0.001617)
Supplement: Uncited Supplementary Material 1. [file mgen-12-01617-s001.pdf]

Supplementary Material (Figures and Methods)

For

Genomic Insights into the Functional and Metabolic Versatility of *Anaerostipes* Species

Disha Bhattacharjee[a], Lindsey C. Millman[a], Meagan L. Seesengood[a], Lindsey M. Martineau[a], and Anna M. Seekatz[a*]

[a]Department of Biological Sciences, Clemson University, Clemson, South Carolina, USA

*Corresponding Author. Life Sciences Building 157A, 190 Collings St, Clemson, South Carolina – 29634, United States of America

Email address: aseekat@clemson.edu

Supplemental Figures and Legends

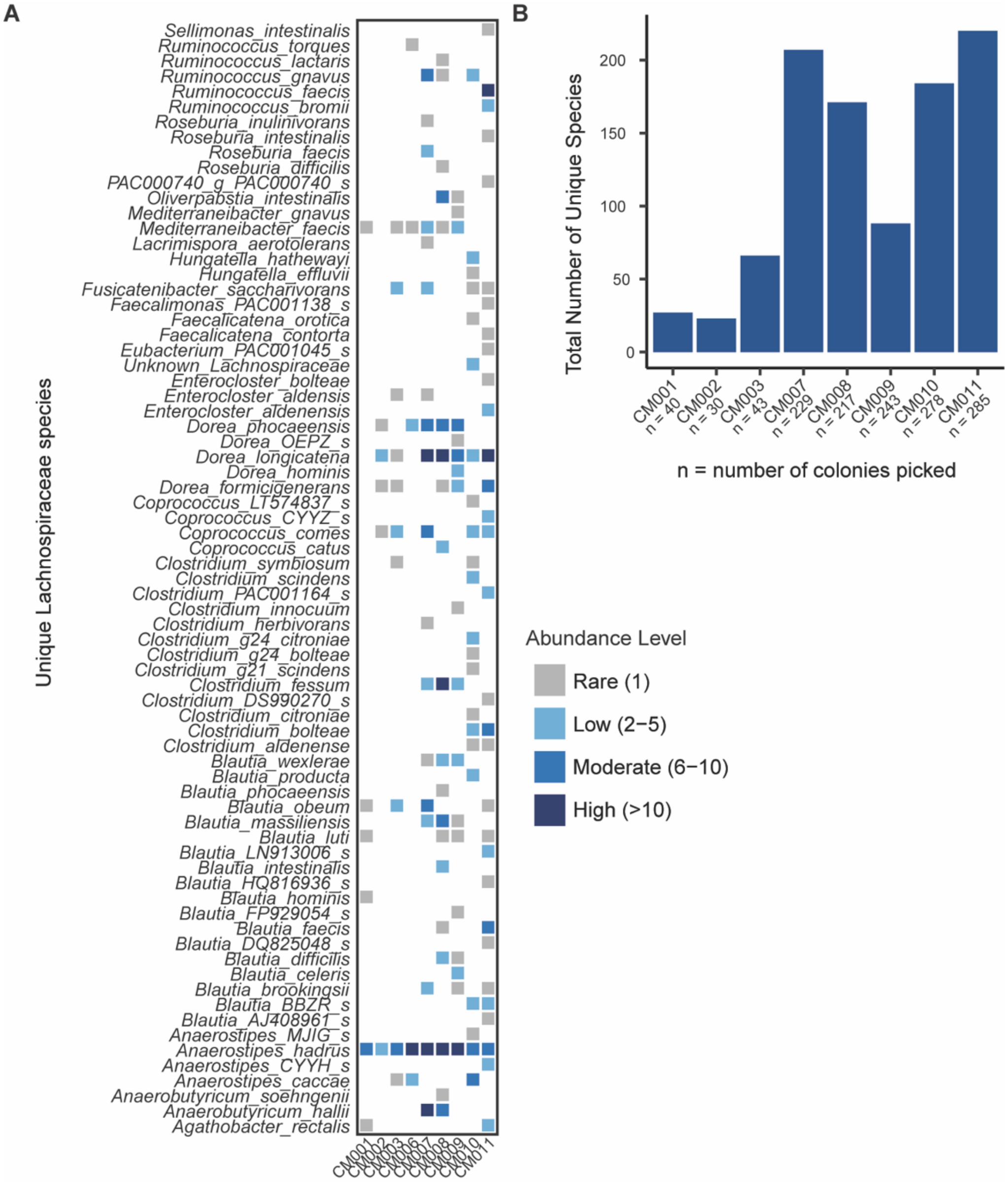

Figure S1. Rescue of unique species increases with the number of colonies picked. A) Number of strains (abundance) of unique Lachnospiraceae species isolated from human fecal samples (subjects on the y-axis). White boxes indicate absence of the strain. **B)** Total number of unique species, across nine human fecal samples (n = number of colonies picked during isolation).

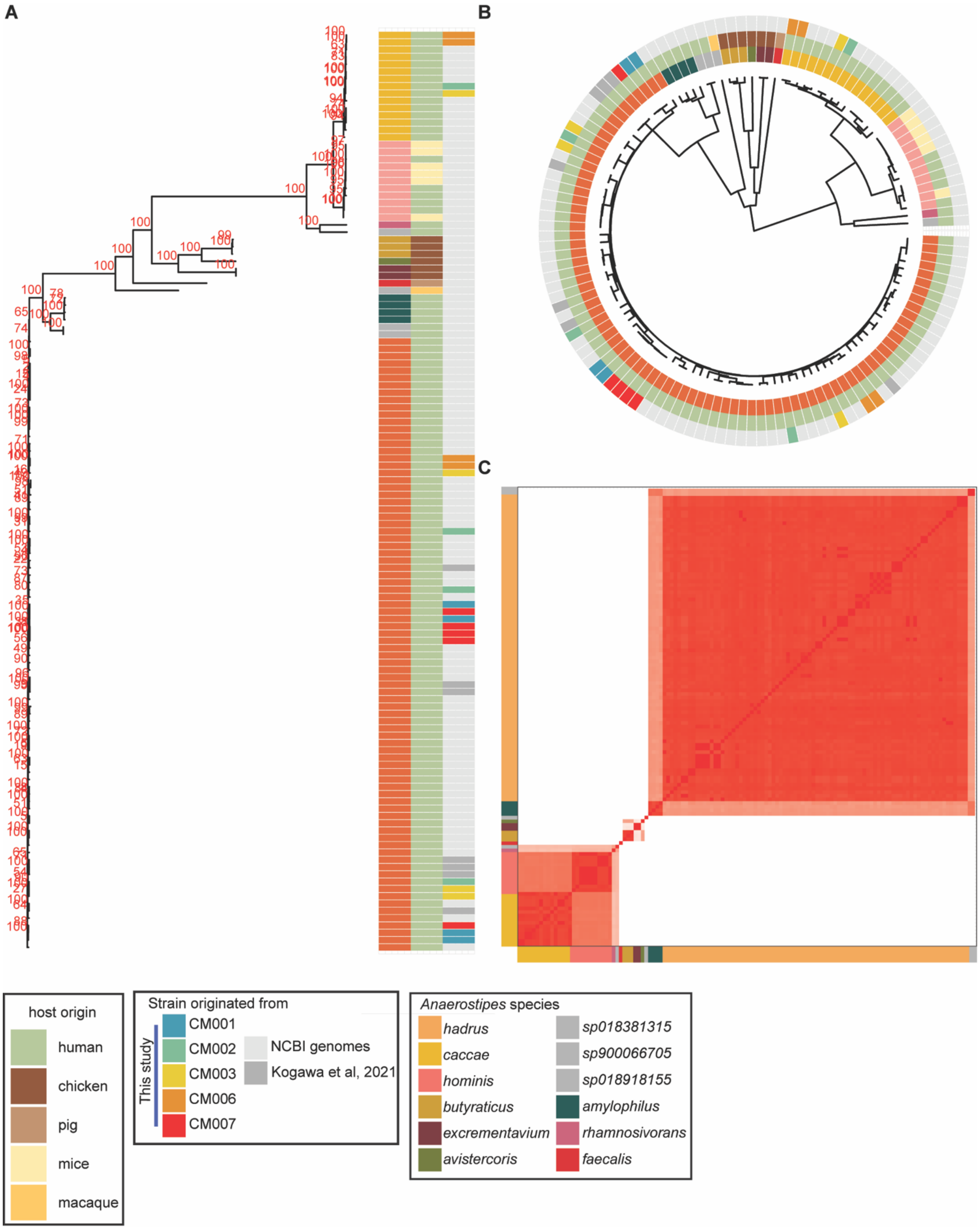

Figure S2. Maximum likelihood trees of *Anaerostipes* based on single nucleotide polymorphisms (SNPs) in the core genome and Average amino acid identity (AAI). A) Maximum likelihood tree made from SNPs in the core genome with bootstrap values in red text

overlayed with species color, host source, and subject. **B)** UPGMA tree of Anaerostipes based on amino acid identity made using ezAAI overlayed with species color, host source, and subject. **C)** Average amino acid identity (AAI) percentage across all 126 genomes, colored by species. (Red denotes 100% ANI; white 80% ANI).

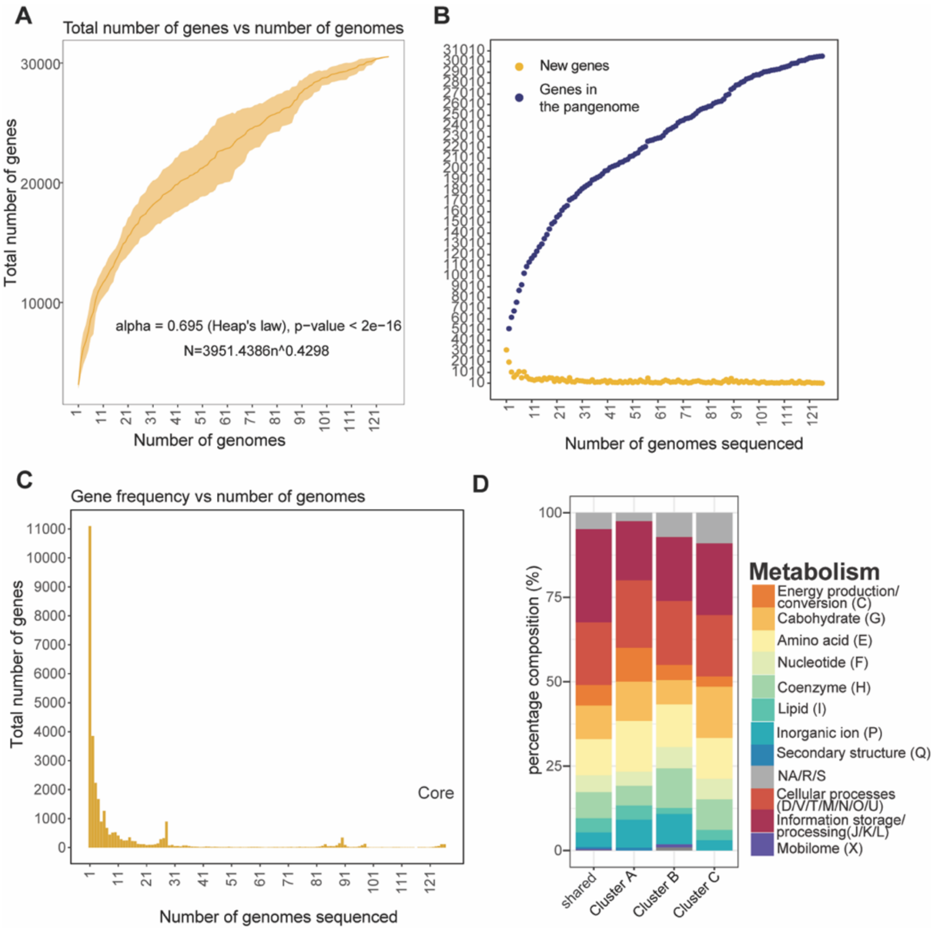

Figure S3. ***Anaerostipes*** **pangenome statistics. A)** Total number of genes as a function of number of all included genomes (n =126). Yellow colored solid line represents the average number of total genes from five subsamplings, with transparent yellow as error bars. **B)** Number of genes as a function of number of genomes depicting number of new genes in yellow dots and number of genes in the pangenome in blue dots of all genomes (n=126). Yellow colored boxplots represent the average number of total genes from five subsamplings. **C)** Total number of unique genes as a function of number of genomes (p-value < 2e^-16). Core genes depicted at 126 genomes by "Core". Alpha = Heap's law estimate, ran over 500 iterations using micropan in R (p-value < 2e^-16). **D)** Relative abundance of COG categories for genes (n=1251) common to each cluster, genes unique to cluster A (n=419), cluster B (n=373), and cluster C (n=132).

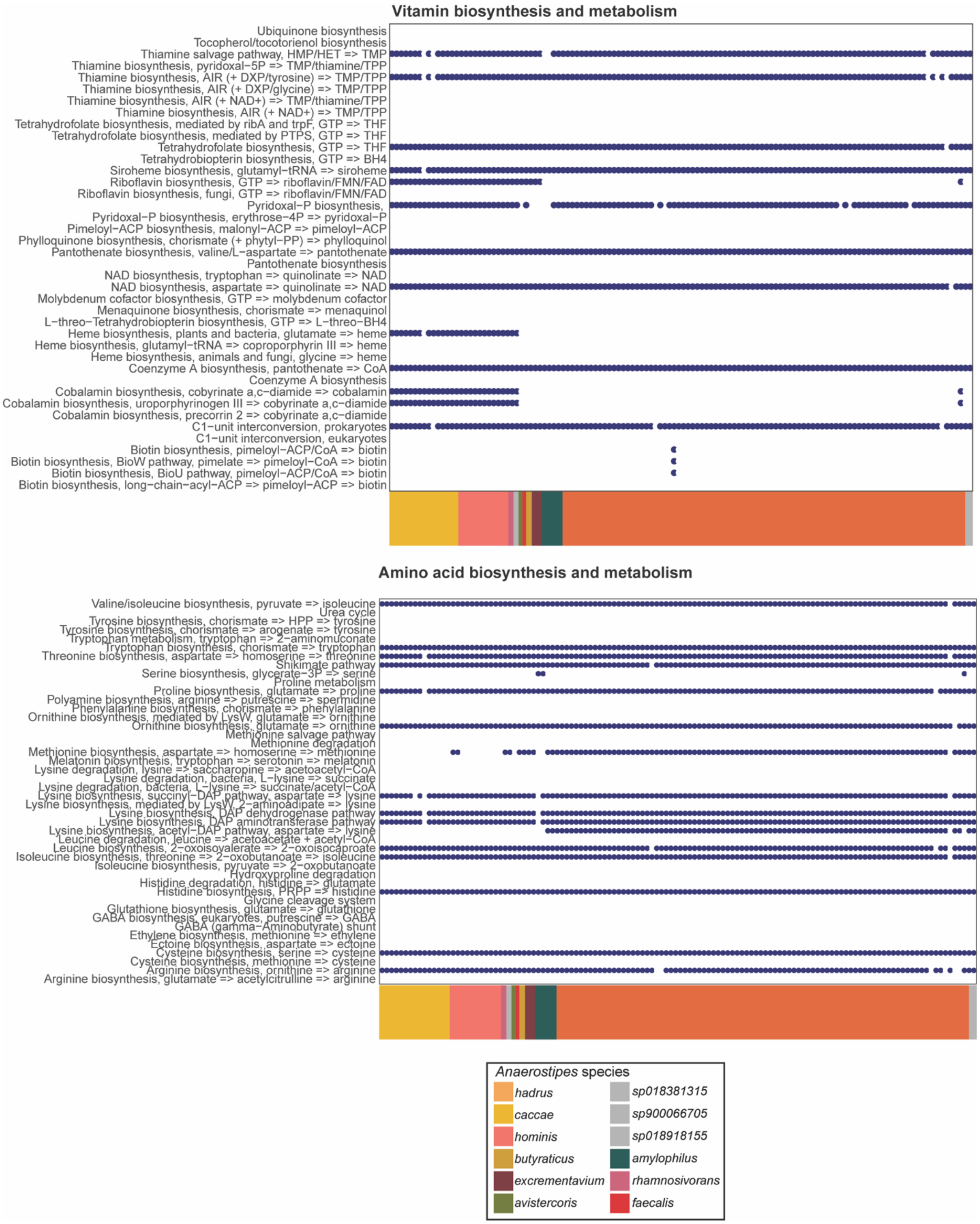

Figure S4. Module completion for amino acid and vitamin biosynthesis and metabolism pathways identified using Anvi'o. The color bar in the x axis depict the species in the legend. Presence is depicted by navy blue circle and absence by white.

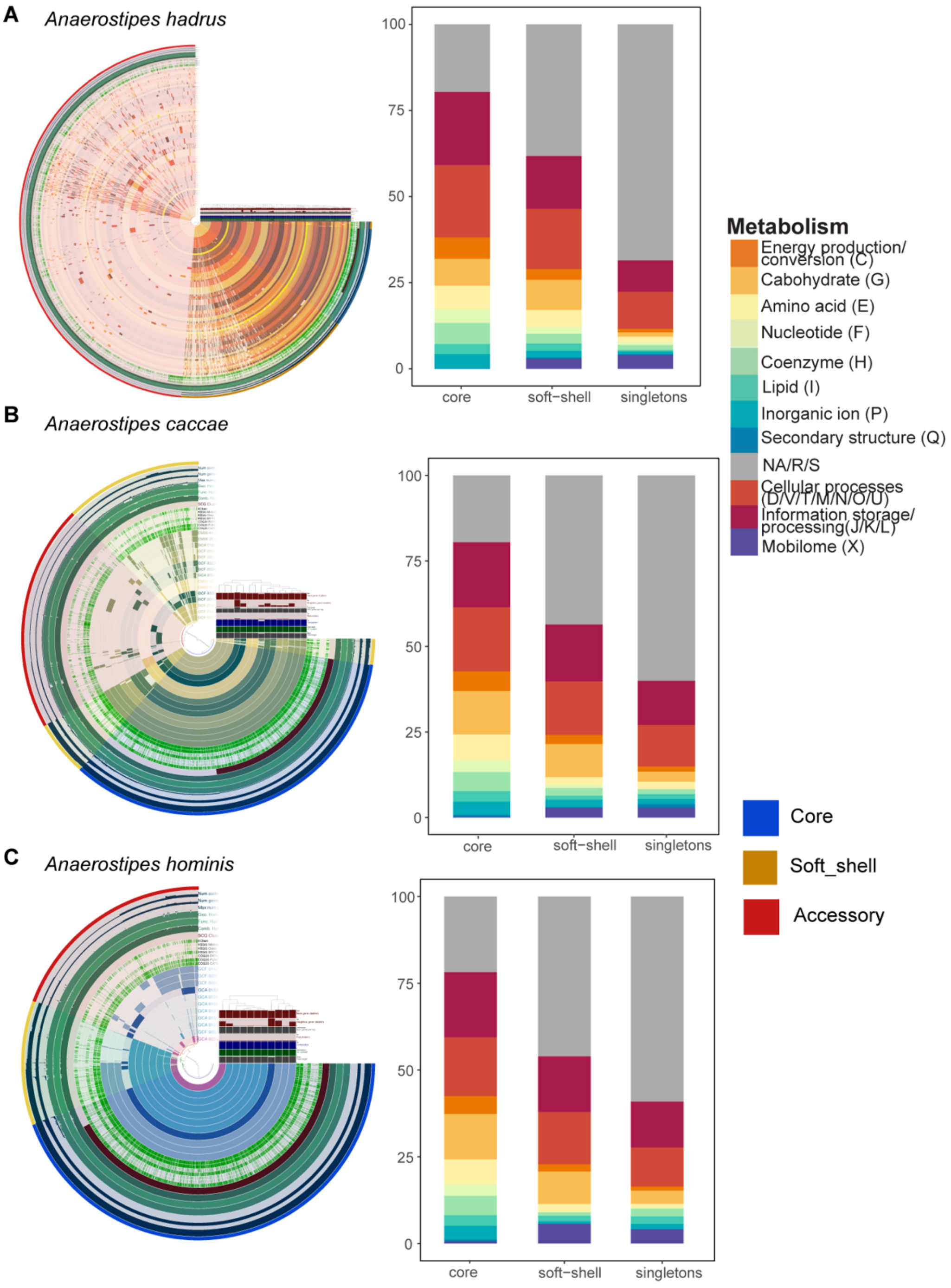

Figure S5. Pangenomes of *A. hadrus*, *A. caccae* and *A. hominis* with the functional COG categorisation of their genes. A) *A. hadrus*, **B)** *A. caccae*, and **C)** *A. hominis* pangenomes created using Anvi'o along with relative percentage of the COG categories the genes belong to, in the core (present in all 100% genomes), soft shell (present in 99% of the genomes) and singleton (present only in a single genome) genes.

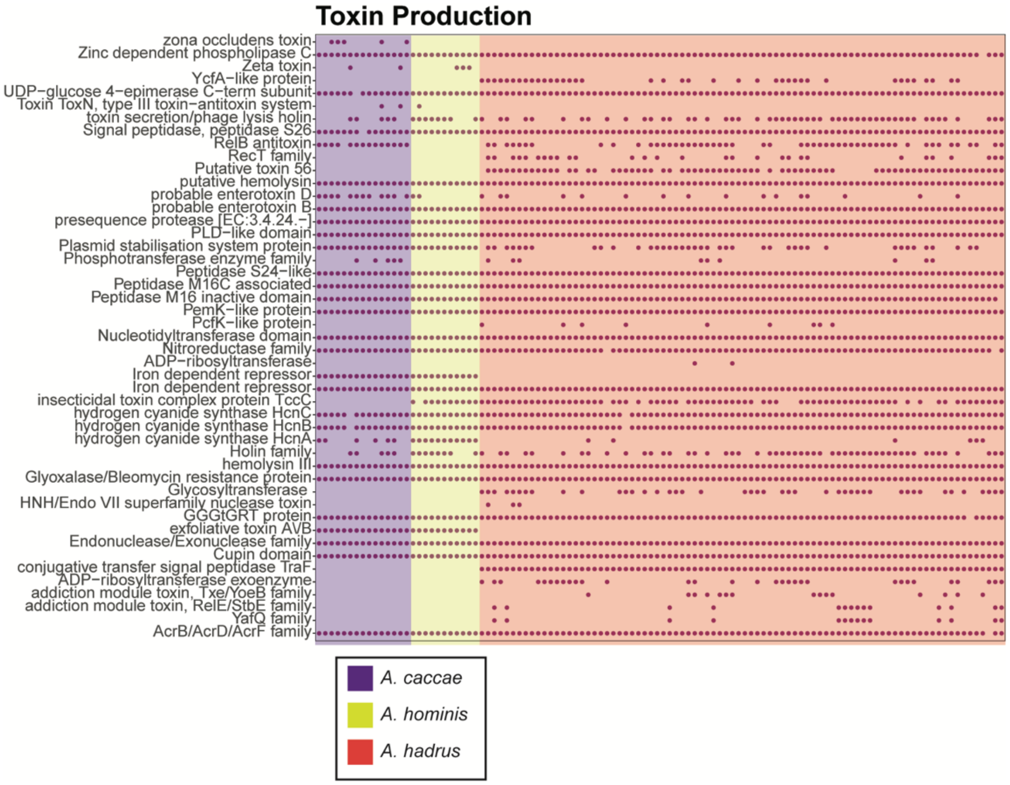

Figure S6. Virulence in *A. hadrus*, *A. caccae* and *A. hominis*. Toxins and virulence factors predicted using PathoFact for *A. hadrus*, *A. caccae*, *A. hominis*.

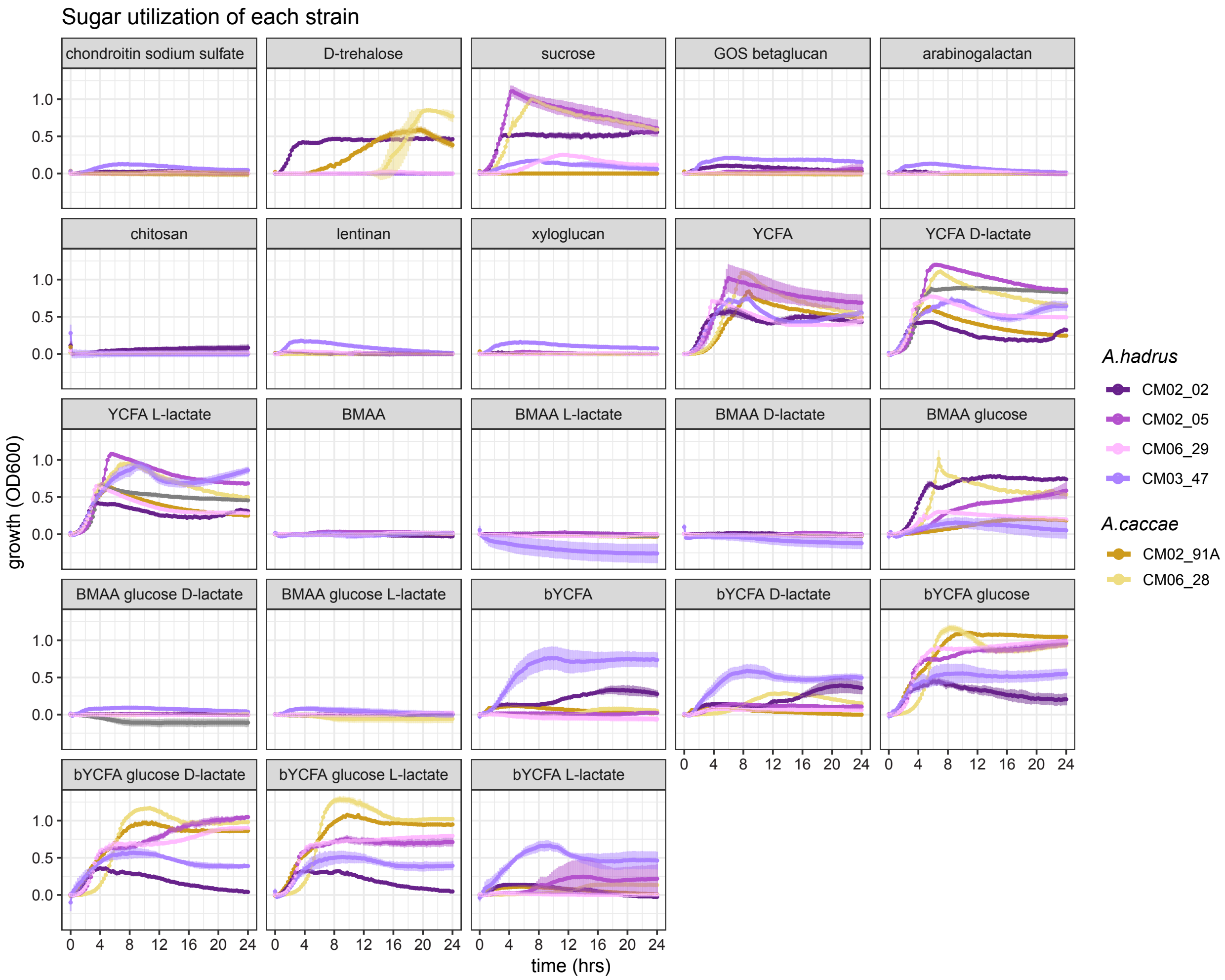

Figure S7. Growth curves of *A. hadrus* and *A. caccae* isolates in BMAA, bYCFA, and YCFA. Isolates were grown in in BMAA, bYCFA, or YCFA supplemented with carbohydrates selected through predicted CAZyme analysis, L-lactate, or D-lactate under anaerobic conditions.

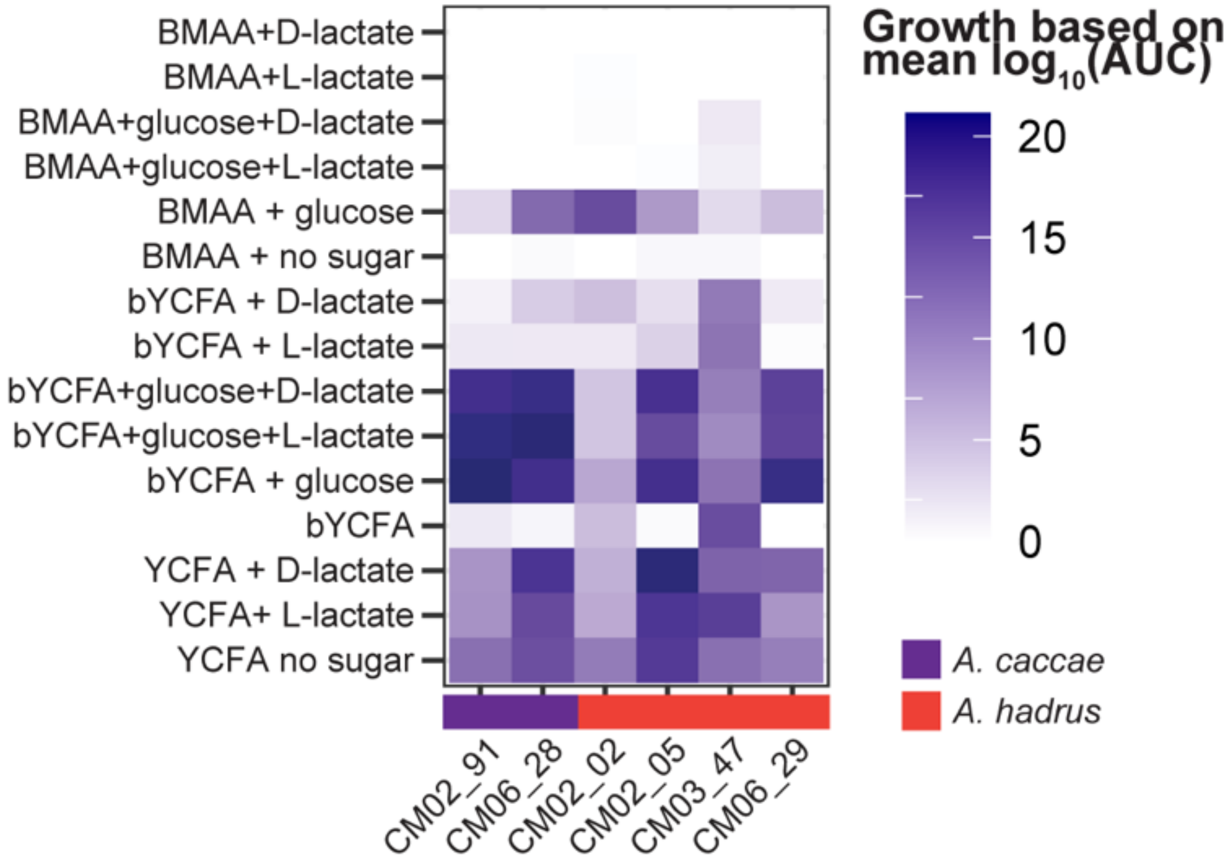

Figure S8. Growth of *A. hadrus* and *A. caccae* denoted by area under the curve (AUC) in different media supplemented with L- or D-lactate. Heatmap of strain growth, as represented by log_{10}(AUC) of optical density (OD_{600}) over time, in minimal media (BMAA), basal (bYCFA), or rich media (YCFA containing glucose and maltose) supplemented with glucose and/or L- or D-lactate.

Supplemental Methods

BMAA Broth – Basal Media with Amino Acids

<u>Materials</u>:

100X Vitamin Stock Solution — see stock prep below
100X Trace Minerals Stock Solution — see stock prep below
100X Trace Elements Stock Solution — see stock prep below
10X Amino Acids Stock Solution — see stock prep below
MilliQ Water
0.1% Resazurin — (Sigma R7017-1G)
TES Sodium Salt — (RPI T31020-100.0)
2 L Erlenmeyer Flask
Aluminum foil
50 mL conical tube — (Fisherbrand 06-443-19)
Sterile 50 mL vacuum filtration system, 0.22micron pore size — (Millipore SCGP00525)

<u>Procedures:</u>

**Ingredient**	**Per 50mL (1X BMAA)**	**Per 50mL (2x BMAA)**
Vitamins	0.5 mL	1 mL
Trace Minerals	0.5 mL	1 mL
Trace Elements	0.5 mL	1 mL
Amino Acids (10X)	5 mL	10 mL
MiliQ Water	Fill up to 50 mL	Fill up to 50mL
0.1% Resazurin	50 µL	50 µL
TES Sodium Salt (buffer) *optional	0.5g	0.5g

1. (for making 50 mL) Obtain 50ml conical tube. After adding ingredients, fill MiliQ water up to 50mL, vortex to mix.
2. Mix well, then filter sterilize with 50ml vacuum filter in the fume hood (0.22micron pore size).
3. Cover tube with foil, and place in chamber at least 1 day before the broth is needed, for adjustment to the anaerobic environment.

Stock Solutions:

Amino Acids (10X), non-sterilized: for 250mL 100X Solution

1. Obtain 2L flask, rinse with MiliQ water. Add all reagents to 250 mL of MiliQ water, add stir bar and mix well. (100X solution)
2. To completely solubilize and dilute down to a 10X solution, add 1.5L of MiliQ water. Heat and stir until completely solubilized.
3. Aliquot into sterile 50mL falcon tubes, store at 4ºC.

Material	Molecular weight (g/mol)	Final Concentration (mM)	Amount (g)
Histidine (A17627.36 Thermo)	155.15	0.644537544	2.5
Tryptophan (Alfa Aesar A10230)	204.23	0.489644029	2.5
Glycine (Alfa Aesar A13816)	75.07	1.332090049	2.5
Tyrosine (Alfa Aesar J63511.22)	181.19	0.551906838	2.5
Arginine (Alfa Aesar A14730)	174.2	1.148105626	5
Phenylalanine (Spectrum chems PH145)	165.19	1.210727042	5
Methionine (Alfa Aesar A10318)	149.21	1.340392735	5
Threonine (Alfa Aesar A16851.22)	119.12	1.678979181	5
Alanine (Alfa Aesar A15804.22)	89.09	2.244920867	5
Lysine (Alfa Aesar A16127.22)	146.19	2.052123948	7.5
Serine (Alfa Aesar A11179.22)	105.09	2.854695975	7.5
Valine (Alfa Aesar J62943)	117.15	2.560819462	7.5
Isoleucine (Alfa Aesar A13699.22)	131.17	2.287108333	7.5
Aspartic Acid (Thermo A13520.22)	133.11	2.253775073	7.5
Leucine (Acros Chems 12512-1000)	131.17	3.049477777	10
Cysteine (Sigma C6852)	240.3	2.080732418	12.5
Proline (Alfa Aesar A10199)	115.13	5.211500043	15
Glutamic Acid (Alfa Aesar A12505)	147.13	6.117039353	22.5

Vitamins (100X), sterilized:

1. Obtain 2L flask, rinse with MiliQ water. Add 500mL of MiliQ water, then reagents. Fill with remaining MiliQ water and mix well.
2. Filter-Sterilize by filtration through a 0.22uM filter tower in the fume hood, cover with foil, and store at 4ºC. For remaining (longer-term) storage, store frozen at -80°C in 50 ml aliquots.

Reagent	Amount per 1L (mg)
Pyridoxine HCl (TCI, P0561)	10
Thiamine HCl (TCI, T0181)	5
Riboflavin (Acros, 132350250)	5
Ca pantothenate (Acros, 243301000)	5

DL-alpha-lipoic acid (TCI, L0058)	5
p-Aminobenzoic acid (Sigma, A9878-5G)	5
Nicotinic Acid (TCI, N0082)	5
Vitamin B12 (Sigma, V6629) (stored at 4C)	5
Mercaptothanesulphonic acid (MESA) (Acros, A0417032)	5
Biotin (Sigma, B4639) (stored at 4C)	2
Folic Acid (Sigma, F7876-1G)	2

Trace Minerals (100X), sterilized:

1. Obtain 2L flask, rinse with MiliQ water. Add 500mL of MiliQ water, then reagents. Fill with remaining MiliQ water and mix well.
2. Filter-Sterilize by filtration through a 0.2uM filter tower in the fume hood, store at room temperature.

Reagent	Amount per 1L (g)
NaCl (Fisherbrand, S271-500)	80
NH4Cl (Alfa Aesar, M14F035)	100
KCl (ACS, 20E0656180)	10
KH2PO4 (Fisherbrand, 202090)	10
MgSO4 * 7H2O (Sigma, SLBZ3736)	20
CaCl2 * 2H2O (Millipore, 208290)	4

Trace Elements (100X), sterilized:

1. Obtain 2L flask, rinse with MiliQ water. Add 500mL of MiliQ water, then reagents. Fill with remaining MiliQ water and mix well.
2. Filter-Sterilize by filtration through a 0.22uM filter tower in the fume hood, store at 4°C.

Reagent	Amount per 1L (g)
Nitriloacetic acid (Alfa Aesar, A18348)	2
Manganese sulfate monohydrate (Acros, 205905000)	1
Ammonium iron(II) sulfate hexahydrate (Acros, 423721000)	0.8
Cobalt(II) Chloride Hexahydrate (C2388)	0.2
ZnSO4*7H2O (Alfa Aesar, A12915)	0.2
Copper(II) chloride dihydrate (Alfa Aesar, 12458)	0.02
NiCl2*6H2O (BTC, 132895-50G)	0.02
Na2MoO4*2H2O (A0410991)	0.02
Na2SeO4 (Alfa Aesar, 12613)	0.02
Sodium tungstate dihydrate (Acros, 424470050)	0.02

Yeast Casitone Fatty Acids Media – YCFA

Materials:

Tryptone	(BD 211059; VWR 90000-060)
Yeast Extract	(BD 212750; VWR 90000-726)
$NaHCO_3$	(Fisher S233-500)
(D)+Glucose	(TCI G0048)
(D)+Maltose	(Sigma-Aldrich M5885-100G)
L-cysteine	(Sigma-Aldrich C6852)
Mineral Solution I (6.7X)	see stock prep
Mineral Solution II (6.7X)	see stock prep
Haemin Solution (100X)	see stock prep
VFA mix	see stock prep
Sodium Acetate	(Sigma-Aldrich S2889-250G)
Sodium Propionic Acids	(Sigma-Aldrich P1880-100G)
Resazurin, 0.1%	(Sigma-Aldrich R7017-1G; see stock prep)
Select agar	(Invitrogen 30391-023; Fisher 30-391-023)
MilliQ water	
NaOH, 4.5N	(Fisher, SS256-500)
Ethanol	

Additional reagent information for stock solutions after recipe

2 1L glass bottles	
25mm syringe filter, 0.22-0.2 micron pore filter size	(Fisherbrand 09-720-004)
30 ml syringe	(BD 302833)

<u>Procedures for 1L agar or broth:</u>

1. (for making 1L) Obtain 2L glass Erlenmeyer flask. Rinse flask with MilliQ water.

2. Fill graduated cylinder with 800mL of MilliQ water. Add 800mL of water to the Erlenmeyer flask. Add stir bar to flask and place on stirring plate.
3. For 1L (or less if making broth), add the appropriate amounts of the following to a 2L flask:

Ingredient	Per 1 L	Final YCFA concentration
Tryptone	10 g	10 g/L
Yeast extract	2.5 g	2.5 g/L
$NaHCO_3$	2 g	2 g/L
(D)+glucose	2 g	2 g/L
(D)+maltose	2 g	2 g/L
L-cysteine	1 g	1 g/L
Mineral Solution I (6.7X)	150 ml	
Mineral Solution II (6.7X)	150 ml	
Haemin Solution (100x)	10 ml	
Sodium acetate	5.58 g	68 mM
Sodium propionic acids	1.8 g	19 mM
0.1% resazurin	1 ml	

4. Stir and mix well with a stir bar and add enough MiliQ water to bring up to 1L (or desired mL amount if making broth).
5. In a fume hood, **add 717 uL of the VFA mix stock (50X)** and mix well. Stir and mix well.
6. Titrate the medium pH by adding 1 mL of 4.5N NaOH dropwise until a pH of 7.45 is reached.
7. If autoclaving broth, split 1 L into 2 500 mL portions in 2 1L glass bottles.
8. Autoclave on cycle 4 (30min @ slow exhaust-liquid). Always use metal autoclave pans with a small layer of water in the bottom. OR: Autoclave (121°C, 60 minutes) on liquid cycle.
9. Remove flask from autoclave and store (covered) in 55°C water bath until cooled (~1 hr).
10. Before aliquoting: For 1 L media, add **1 ml Vitamin Stock solution (1000X)** to the media.
    a. If making broth, add 0.5 mL into each 500 ml bottle.
11. Ensure that bottle is cooled down completely, tighten cap, and store in cold room at 4°C.

<u>Stock solutions</u>

Mineral solution I (6.7X), non-sterile:

1. Add reagents to 1 L milliQ water; store at room temperature.

Reagent	Amount per 1L (g)	Concentration in YCFA
K_2HPO_4 (product number)	3	0.45 g/L

Mineral solution II (6.7X), non-sterile:

1. Add reagents to 1 L milliQ water; store at room temperature.

Reagent	Amount per 1L (g)	Concentration in YCFA
K_2HPO_4 (Fisherbrand, 202090)	3	0.45 g/L
NaCl (Fisherbrand, S271-500)	6	0.9 g/L
$(NH_4)_2SO_4$ (Millipore, AX1385)	6	0.9 g/L
$MgSO_4$ x $7H_20$ (Sigma, SLBZ3736)	0.6	0.09 g/L
$CaCl_2$ x $2H_20$ (Millipore, 208290)	0.6	0.09 g/L

Haemin Solution (100X; 1 mg/ml), non-sterile:

1. Dissolve the (below) KOH in ethanol.
2. Then add haemin (below). When dissolved, add water.

Reagent	Amount per <u>100 mL</u>	Concentration in YCFA
KOH (Fisher P250-500)	0.28 g	
Ethanol	24 ml	
Haemin (Sigma H9039)	100 <u>mg</u>	1 mg/L
Deionized water	75 ml	

Resazurin (0.1%), non-sterile:

1. Add reagent and dissolve; wrap tube in foil and store at 4°C.

Reagent	Amount per 50 ml	Concentration in YCFA
Resazurin (Sigma R7017-1G)	0.05 g	0.001 g/L
MilliQ water	50 ml	

VFA Mix (Fatty Acid Mix, 50X), non-sterile:

1. In fume hood, add the following short chain fatty acids.

Reagent	Amount per <u>30 mL</u>	Concentration in YCFA

Isobutyric acid (I1754)	10 ml	1 mM
Valeric acid (Sigma-Aldrich, 240370)	10 ml	1 mM
Isovaleric acid (Sigma-Aldrich, 129542)	10 ml	1 mM

Vitamin Mix (1000X), sterilized:

1. Stir all reagents together until dissolved.
2. Titrate with 4.5N NaOH (0.5-2 drops) to pH 7.0.
3. Filter-sterilize through a 0.22uM filter bottle.
4. Aliquot into ~50 ml portions in 50 ml falcon tubes. For working tube, store in the dark (cover with foil) at 4°C. For remaining (longer-term) storage, store frozen at -80°C.

Reagent	Amount per 500 ml	Concentration in YCFA
Biotin (Sigma, B4639)	5 mg	10 µg/L
(cyano)colbalamin (B12) (Sigma, V6629)	5 mg	10 µg/L
4(para)-aminobenzoic acid (Sigma, A9878-5G)	15 mg	30 µg/L
Folic acid (Sigma, F7876-1G)	25 mg	50 µg/L
Pyridoxine HCl (TCI, P0561)	75 mg	10 µg/L
Thiamine HCl (TCI, T0181)	25 mg	50 µg/L
Riboflavin (Acros, 132350250)	25 mg	50 µg/L
Deionized water	1 L	
